# Potential‐Dependent Kinetics and Reaction Pathways of Low‐Potential Furfural Electrooxidation with Anodic H_2_ Production

**DOI:** 10.1002/smsc.202500132

**Published:** 2025-06-24

**Authors:** Zhaohui Wu, Guihao Liu, Ziheng Song, Yihang Hu, Tianqi Nie, Yu‐Fei Song

**Affiliations:** ^1^ State Key Laboratory of Chemical Resource Engineering Beijing University of Chemical Technology Beijing 100029 P. R. China; ^2^ Quzhou Institute for Innovation in Resource Chemical Engineering Quzhou Zhejiang Province 324000 P. R. China

**Keywords:** anodic H_2_ production, furfural electrooxidation, oxide‐derived copper, potential‐dependence

## Abstract

The low‐potential furfural electrooxidation reaction (FFOR) on copper‐based catalysts provides a novel pathway to upgrade biomass and produce H_2_ simultaneously on anode. Herein, a series of oxide‐derived copper catalysts (OD‐Cu‐x, x represents electroreduction time) with distinct Cu^0^/Cu^+^ ratios and residual content of lattice oxygen are successfully constructed by tuning in‐situ electroreduction time. When applied for FFOR, the OD‐Cu‐600 with a Cu^0^/Cu^+^ ratio of 83.3% shows the Faradaic efficiency of 96.1% for furoic acid (FA) and 97.4% for H_2_, which can be achieved at a lowest potential of 0.081 V versus RHE at 10 mA cm^−2^ in continuous 10 cycles, outperforming the state‐of‐art Cu‐based catalysts reported so far. Detailed characterization and density functional theory (DFT) calculations prove that the moderate coverage (25% based on DFT models) of Cu(OH)_ads_ surface species generated by Cu^+^ during the electrooxidation process endows the optimal furfural molecule adsorption and activation. Moreover, this potential‐dependent coverage of surface OH can promote the kinetics of *H transfer to the Cu surface, allowing the H_2_ evolution from the anode. The Cu^0^/Cu^+^ ratio (83.8%) and suitable applied potential windows (0 to 0.4 V *vs* RHE) are both responsible for the co‐production of FA and H_2_ with high intrinsic activity and efficient H atom utilization.

## Graphical abstract

The adsorption behavior of furfural molecules and kinetics of *H transfer were both modulated by potential‐dependent coverage of (OH)_ads_, and the suitable potential window (0‐0.4 V vs RHE) was selected for the co‐production of furonic acid and H_2_ with optimal activity and high Faradaic efficiency.

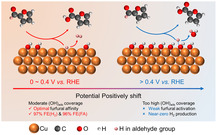

## 1 Introduction

**Scheme 1:**
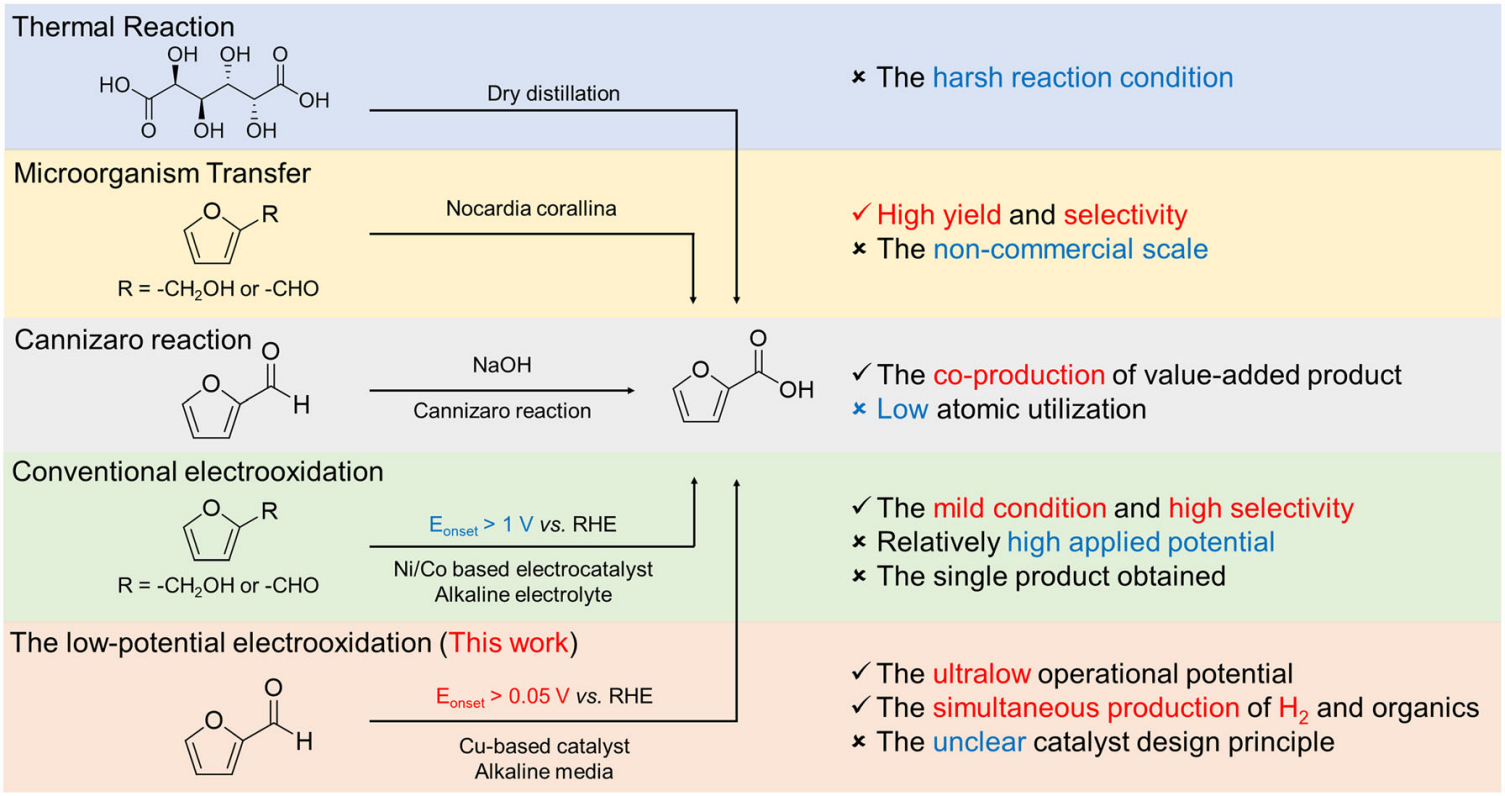
The comparison of FA synthesis routes.

In the context of energy crisis, H_2_ is seen as a promising future energy source. Coupling organics electrooxidation with cathodic hydrogen evolution reaction (HER) has been proven as a promising strategy and an environmentally friendly route to produce value‐added organics (anode) and H_2_ (cathode) simultaneously.^[^ ^1^, ^2^, ^3^, ^4^ ^]^ However, the onset and operating potential of this type of electrooxidation reaction are both relatively high (usually > 1 V *vs* RHE), and the hydrogen atoms in the substrate molecules tend to react with OH^−^ in the alkaline electrolyte via a reverse Volmer process (*H + OH^−^ = H_2_O + e^−^) to produce valueless H_2_O, reducing the atomic utilization efficiency.^[^ ^5^, ^6^, ^7^, ^8^ ^]^ Surprisingly, van den Meerakker found that formaldehyde (HCHO) can be converted to formate and produce H_2_ at anode simultaneously on Cu electrodes.^[^ ^9^ ^]^ Wang et al.^[^ ^10^ ^]^ investigated the novel aldehyde oxidation process and proposed that the H atom in the aldehyde group can transfer to the surface and transformed to H_2_ instead of H_2_O since the H adsorption energy (E_ads_) of near zero.^[^ ^11^ ^]^ After that, the low‐potential aldehyde oxidation reaction became a novel anodic half‐reaction candidate for direct fuel cells.^[^ ^12^, ^13^, ^14^ ^]^ Moreover, the high energy efficiency and the value‐added bipolar H_2_ production enabled the formation of FA, an important feedstock in food and medicine industry, via a more energy‐efficient and atom‐economic pathway than other conventional routes (**Scheme** **1**). However, the absence of in‐depth investigation for structure–performance relationships casts a shadow over the design of efficient catalysts for low‐potential aldehyde reactions.

The transition metal‐based, especially copper‐based catalyst, was widely utilized in electrocatalytic systems such as the electroreduction of CO_2_ and biomass upgrading,^[^ ^15^, ^16^, ^17^, ^18^ ^]^ and the active sites induced by structure evolution under reaction conditions usually played an important role in the performance.^[^ ^19^, ^20^, ^21^ ^]^ Wang et al.^[^ ^22^ ^]^ found that mix‐value Cu (MV‐Cu) exhibited an obviously higher furfural electrooxidation activity than Cu and Cu_2_O, but further electrooxidation of the catalyst under anodic potential inhibited the affinity for furfural molecules. Moreover, the investigation of potential‐dependent transformation of intermediate, which considers the influence of applied potential, has become a hot spot in the field of electrocatalysis and can help to explore the reaction pathway.^[^ ^23^, ^24^, ^25^ ^]^ Luo et al.^[^ ^26^ ^]^ found that the H atom of aldehyde in 5‐hydromethylfurfural (5‐HMF) can transfer to the surface of catalyst as a hydride (H^−^) and desorbed to form H_2_ under 0.13–0.43 V versus RHE, while the H atom with positively charged can only be detached by OH^−^ and produce H_2_O in the range of 0.13–0.93 V versus RHE. However, the dynamic evolution process and real microenvironment of Cu‐based catalysts under the condition of low‐potential furfural electrooxidation reaction (FFOR) have not been deeply explored and investigated in detail.

Herein, a series of oxide‐derived copper (OD‐Cu‐x, x representing the in‐situ electroreduction time) catalysts, featuring distinct Cu^0^/Cu^+^ ratios and microenvironments with residual lattice oxygen, were successfully prepared via a valence engineering strategy. The OD‐Cu‐600 with a moderate Cu^0^/Cu^+^ ratio exhibited a superior activity with only a potential of 0.081 V versus RHE in furfural electrooxidation. On the anode, the FA and H_2_ can be produced with the FE of 96.1% and 97.4%, respectively, and the performance can be maintained in 10 consecutive tests. The comprehensive characterization and theoretical simulations confirm that the furfural adsorption behavior and *H transfer pathway were both modulated by the potential‐dependent coverage of Cu sites with hydroxyl adsorption (Cu(OH)_ads_, which was obtained from the transformation of Cu^+^ site under alkaline conditions and the potential‐dependent adsorption of OH^−^ in anolyte). Thus, the moderate Cu^0^/Cu^+^ ratio and suitable potential window (0 to 0.4 V vs RHE) were simultaneously required for efficient co‐production of FA and H_2_ on the anode.

## 2 Results and Discussions

### 2.1 Material Synthesis and Characterization

**Figure 1:**
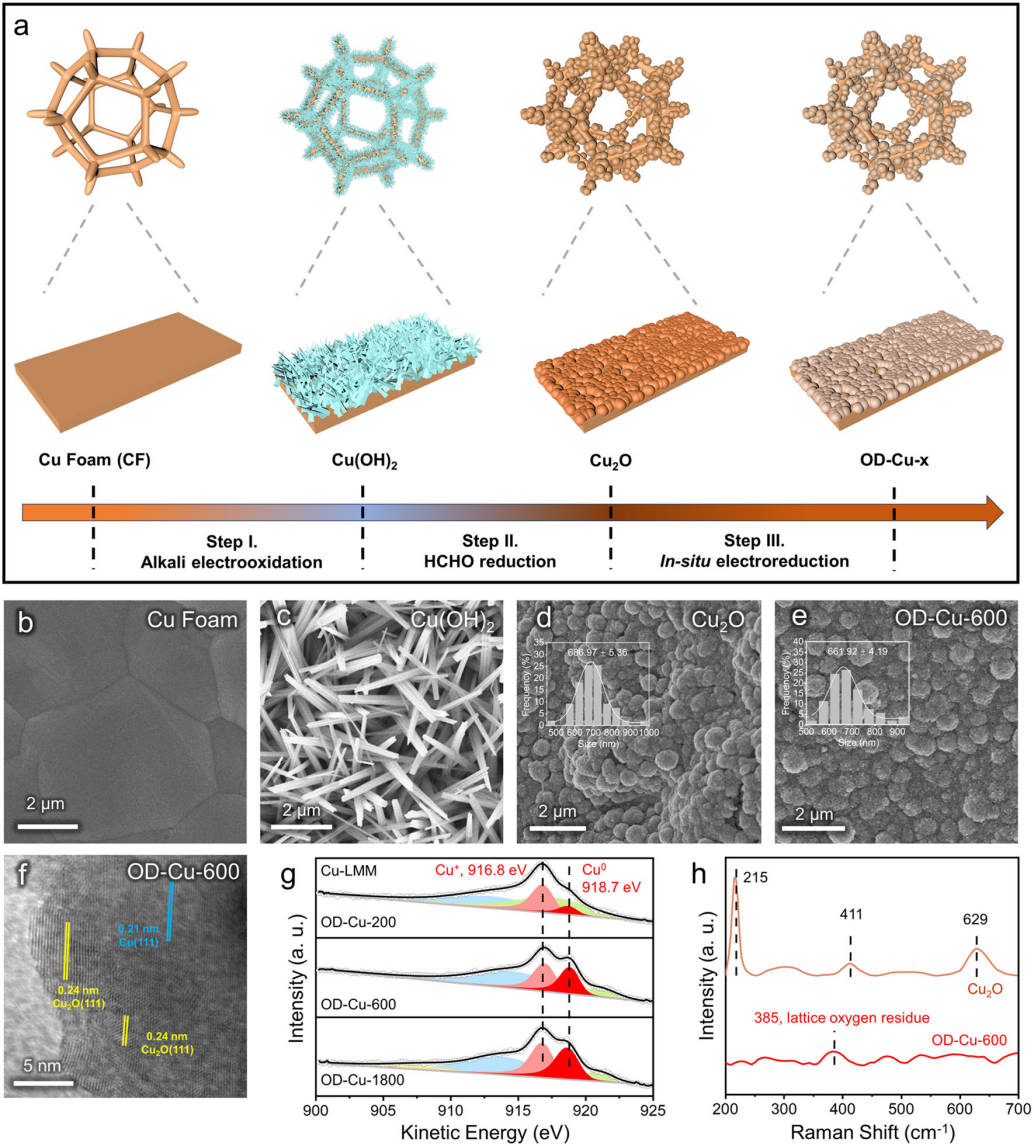
Catalyst synthesis and morphology characterization. a) The schematic illustration for the synthesis procedure of OD‐Cu‐x catalysts; b–e) the SEM images of Cu foam, Cu(OH)_2_, Cu_2_O and OD‐Cu‐600, respectively, the inset plot in (c) and (d) was the size distribution of Cu_2_O and OD‐Cu‐600; f) the HRTEM images of OD‐Cu‐600; g) the fitting of Cu LMM spectra of OD‐Cu‐x catalysts (x = 200, 600, 1800); h) the Raman spectra of Cu_2_O and OD‐Cu‐600.

The OD‐Cu‐x catalyst was prepared with a process including electrooxidation, wet chemical reduction, and in‐situ electroreduction (**Figure** **1**a).^[^ ^27^, ^28^ ^]^ During the electrooxidation process of Cu foam, the chronoamperometry (CP) curve showed an obvious upshift of potential, corresponding to a dominant reaction switched from Cu^0^/Cu^+^‐to‐Cu^2+^ to oxygen evolution reaction (OER). Thus, the electrooxidation was stopped immediately when the potential plateau appeared (Figure S1, Supporting Information). The obtained material was confirmed as Cu(OH)_2_ by X‐ray diffraction (XRD) test. After the formaldehyde (HCHO) reduction, Cu(OH)_2_ was completely transformed to Cu_2_O precursor. Compared with the bare Cu foam (no peaks in the range of 3≈40°), the characteristic peak of Cu(OH)_2_ (PDF #13‐0420) and Cu_2_O (PDF #05‐0667) appeared after the process described earlier, indicating the successful synthesis of Cu(OH)_2_ and Cu_2_O (Figure S2).

A series of scanning electron microscopy (SEM) was employed to monitor the morphology transformation during the synthesis procedure. The Cu(OH)_2_ with typical nanorod morphology grew on the smooth and flat surface of Cu foam (Figure 1b,c, and Figure S3). Besides, the Cu_2_O showed a rough sphere appearance with an average diameter of ≈686 nm (Figure 1d). This morphology and size were conserved after the in‐situ electroreduction, with the OD‐Cu‐600 exhibiting a spherical shape and an average size of ≈661 nm (Figure 1e). The fine structure was characterized by high‐resolution transmission electron microscopy (HRTEM). In Figure 1f, the OD‐Cu‐600 exhibited the lattice fringe spacing of 0.24 nm and 0.21 nm, which can correspond to Cu_2_O(111) and Cu(111), respectively. The morphologies of OD‐Cu‐200 and OD‐Cu‐1800 were also explored. The average grain sizes of OD‐Cu‐200 and OD‐Cu‐1800 were ≈680 nm and ≈674 nm, respectively. The lattice fringes of Cu_2_O(111) and Cu(111) are also shown in the HRTEM image. These results excluded the morphological difference among OD‐Cu‐x catalysts (Figure S4).

The OD‐Cu‐x (x = 200, 600, 1800) catalysts with different surface Cu^0^/Cu^+^ ratios were obtained by controlling the in‐situ reduction time, and the electronic structure and surface species were deeply studied with spectroscopy. The Cu LMM and XPS Cu 2p spectra were tested to identify the different oxidation states of OD‐Cu‐x (Figure 1g and Figure S5). The Cu^2+^ peak did not appear in Cu LMM and Cu 2p spectra, manifesting the complete reduction of Cu^2+^. There were two peaks located at 916.8 eV and 918.6 eV, corresponding to the Cu^+^ and Cu^0^, respectively. According to the fitting results of Cu LMM, the ratios of Cu^0^/Cu^+^ were 24.0% in OD‐Cu‐200, 83.3% in OD‐Cu‐600, and 94.2% in OD‐Cu‐1800, which were different (Table S1). In O 1s spectra, the lattice oxygen (related to the peaks at ≈531eV) reduced from 23.4% (in Cu_2_O) to 15.4% (in OD‐Cu‐600) after the electroreduction. By comparison, the ratios of OD‐Cu‐200 and OD‐Cu‐1800 were 19.2% and 15.1%, respectively, manifesting the microenvironment with residual lattice oxygen in all OD‐Cu‐x catalysts (Figure S6, Table S2). Raman spectrum was employed to uncover the microstructure. The peaks located in 215, 411, and 629 cm^−1^ can be correlated to the second‐order Raman‐allowed mode, four‐phonon mode (3Γ^−^_12_ + Γ^−^_25_), and infrared‐allowed vibration of mode in Cu_2_O, respectively.^[^ ^29^, ^30^, ^31^ ^]^ After the in‐situ electroreduction, all previous peaks disappeared, leaving only a peak at 385 cm^−1^, which can be assigned to the multi‐phonon process associated with the residual lattice oxygen in copper, indicating the microenvironment with the lattice oxygen anchored.^[^ ^32^ ^]^ (Figure 1h) Based on the aforementioned characterization, the polycrystalline OD‐Cu with a mixture of Cu^0^ and Cu^+^ and the residual lattice oxygen was validated.

### 2.2 The Electrochemical FFOR Performance

**Figure 2:**
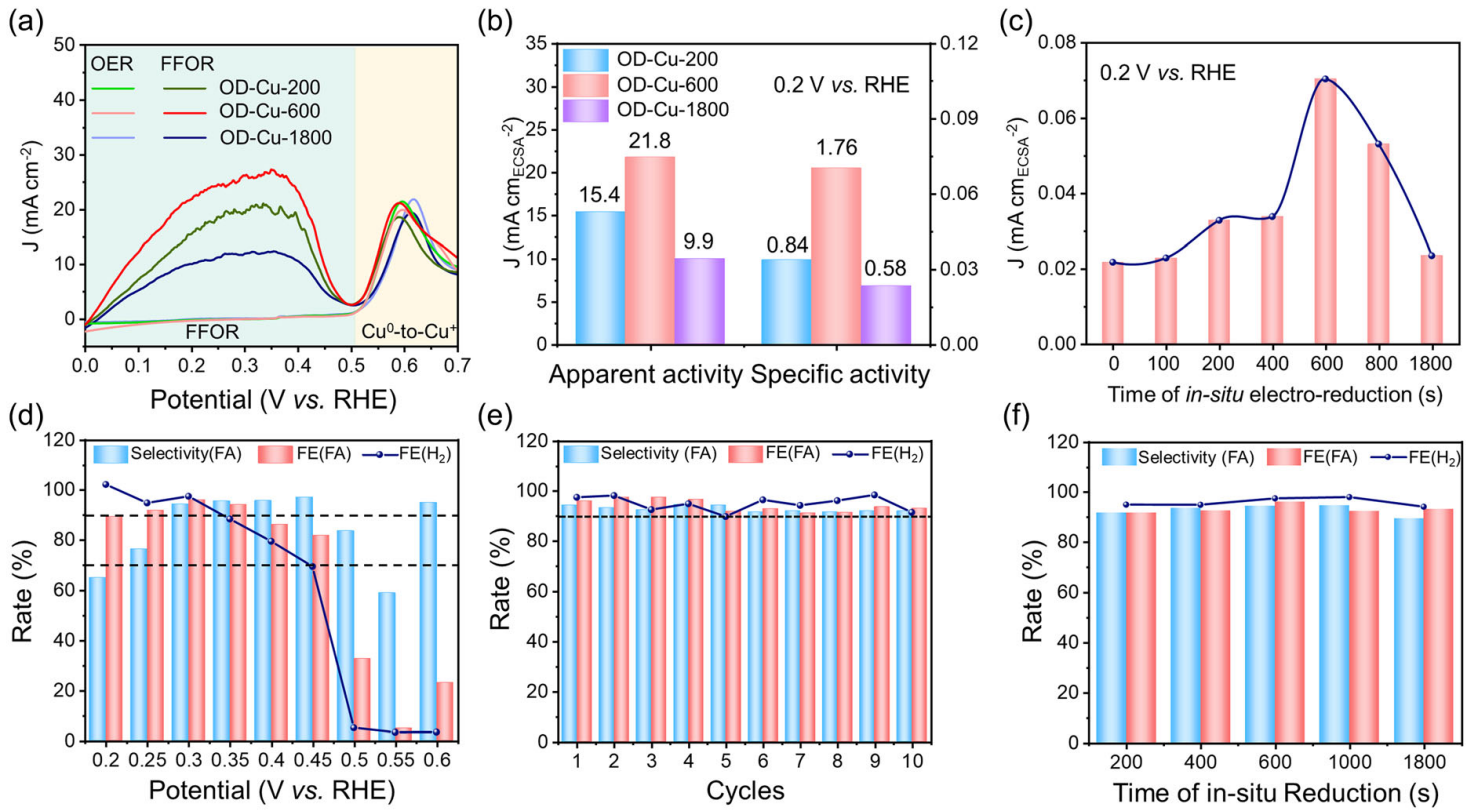
The FFOR performance of various catalysts. a) The LSV curves of OD‐Cu‐x (x = 200, 600, and 1800) in 1 M KOH with/without 50 mM FF; b) the comparison of apparent activity and specific activity among the OD‐Cu‐200, OD‐Cu‐600, and OD‐Cu‐1800; c) the specific activity of OD‐Cu‐x catalyst obtained with different in‐situ electroreduction; d) the selectivity of FA, the FE of FA and anodically produced H_2_; e) the cycle test; f) the FE and selectivity of anodic products.

The FFOR performance of as‐prepared OD‐Cu‐x catalysts was tested in a typical three‐electrode system with an electrolyte of 1 M KOH. The linear sweep voltage (LSV) curves showed negligible current until 0.5 V versus RHE, at which point an obvious oxidation peak emerged, corresponding to a Cu^0^‐to‐Cu^+^ electrooxidation process. After 50 mM FF was added to the anolyte, the current density of three OD‐Cu catalysts exhibited a significant increase during the potential window from 0 to 0.5 V versus RHE, corroborating that they all displayed activity for FFOR. The activity of bare Cu foam was also compared with OD‐Cu‐x (Figure S7). The apparent and intrinsic activity of bare Cu foam were both significantly lower than OD‐Cu‐x, revealing that the OD‐Cu‐x catalyst was the active component. The low‐potential FFOR process occurred before the oxidation of Cu^0^, and the current density was hardly higher than that in 1 M KOH after the Cu^0^‐to‐Cu^+^ peaks at ≈0.5V versus RHE, both suggesting that Cu^0^ can be the active state of FFOR. The OD‐Cu‐600 delivered the best activity among the three catalysts, only a potential of 0.081 V versus RHE was needed to reach a current density of 10 mA cm^−2^, outperforming most state‐of‐art electrocatalysts for low‐potential furfural electrooxidation (**Figure** **2**a, Table S3).

To highlight the changes in the intrinsic activity of three OD‐Cu catalysts, the capacities of double layer (C_dl_) were measured, and the activity was normalized with ECSA to compare the intrinsic activity (Figure 2b, Figure S8). The OD‐Cu‐600 exhibited the highest inherent activity of 0.070 mA cm_ECSA_^−2^, which was significantly higher than the OD‐Cu‐200 (0.034 mA cm_ECSA_^−2^) and OD‐Cu‐1800 (0.023 mA cm_ECSA_^−2^). For screening the optimal time of in‐situ electroreduction and obtaining the most superior catalyst, a series of OD‐Cu catalysts were prepared through different electroreduction times, followed by the calculation of their intrinsic activity. Among them, the OD‐Cu‐600 exhibited the highest intrinsic activity (Figure 2c, Figure S9), suggesting that the different Cu^0^/Cu^+^ ratio endowed by different times of in‐situ electroreduction had an apparent influence on the intrinsic activity of OD‐Cu‐x catalysts.

The anodic products of OD‐Cu‐600 were collected and quantified to examine the selectivity and FE for the FFOR (Figure 2d, Figure S10). In the potential window from 0.2 to 0.35 V versus RHE, the FE of FA and H_2_ remained above 90%. The relatively low selectivity at 0.2 and 0.25 V versus RHE can be attributed to the low current density and the inevitable Cannizzaro reaction (2 R‐CHO + OH = R‐COO^−^ + R‐CH_2_OH), resulting in the non‐Faradaic process consuming a significant amount of FF and producing furfuryl alcohol (FL). The highest selectivity of FA (94.4%) and FE of FA (96.1%) and H_2_ (97.4%) were achieved under the 0.3 V versus RHE. It was worth noting that the FE of products presented a significant difference in the distinct potential ranges. When the operative potentials were higher than 0.4 V versus RHE, the FE of H_2_ represented a significant decrease, with a value of 79.4% at 0.4 V versus RHE and 69.3% at 0.45 V versus RHE, respectively. Furthermore, the H_2_ production process was almost totally expressed when the potential came to higher than 0.5 V versus RHE. The yield rate of FA and H_2_ can also show the trend (Figure S11). The FA and H_2_ can be produced on anode stoichiometrically, and the ratio relationship broke after 0.35 V versus RHE. To understand the decay of selectivity and FE, the possible overoxidation products (e.g., CO_2_ and maleic acid) were also tested. The gas chromatograph (GC) confirmed that there is no CO_2_ formation during reaction and the ^1^H‐NMR showed that no maleic acid (characteristic peak in *δ* = 5.87 ppm) appeared, suggesting that the FF has not been overoxidized (Figure S12, S13). These results hinted that the reaction pathway of FFOR can vary in the different potential windows. In the 10 consecutive cycle tests under this optimal potential, the selectivity of FA and the FE of FA and H_2_ were all maintained above 90%, manifesting the superior catalytic stability of OD‐Cu‐600. The anodic product distributions of OD‐Cu‐x (x = 200, 400, 100, 1800) were also quantified (Figure 2f). The selectivity and FE of FA and H_2_ had an unnoticeable nuance with OD‐Cu‐600, indicating that the main FFOR performance difference of OD‐Cu‐x catalysts focused on the intrinsic activity rather than product distribution.

### 2.3 The Potential‐Dependent Kinetics Transition of OD‐Cu‐x Catalysts

The activity origin of OD‐Cu‐x catalysts was first investigated. According to the LSV curves in Figure 2a, the current density of FFOR had no significant increase than that after the formation of Cu^+^ at ≈0.5V versus RHE. Moreover, when 20 mM KSCN (the poisoning agent of Cu^0^) was added to the electrolyte, the current of the FFOR reaction became hardly detectable (Figure S14).^[^ ^33^ ^]^ These phenomena suggested that the Cu^0^ site was the active site, and Cu^+^ species was inactive for low‐potential FFOR.

**Figure 3:**
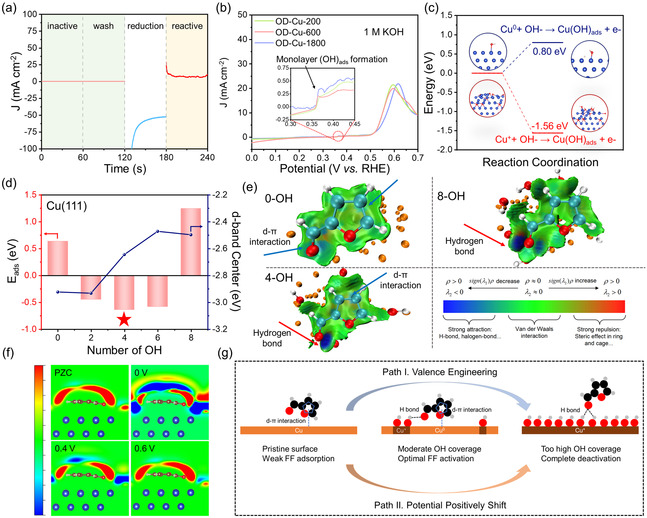
The investigation of the activity origin of OD‐Cu‐x catalysts. a) The i–t curve during the activity recovery process of OD‐Cu‐600 catalyst; b) the LSV curves of OD‐Cu‐x (x = 200, 600, 1800) in 1 M KOH, the inset plot was the oxidation peaks under 0.37 V versus RHE; c) the energy profile of Cu^0^ and Cu^+^ transfer to Cu(OH)_ads_; d) the adsorption energy of furfural on the Cu(111) with different OH coverage and the Cu 3 d band center of Cu(111) with different OH covered; e) the contour plot of IGMH analysis on the Cu(111) with zero, four, and eight OH covered, the corresponding region of weak interaction was represented with the color bar; f) the 2D contour plot of electron density for FF adsorption on under different potential, the range of color bar is ±5 × 10^−3^ e Bohr^−3^, g) the schematic illustration of the effect of (OH)_ads_ coverage for the FFOR activity on Cu(111).

Furthermore, during the cycling test, it was noteworthy that after a single test, the electrode had a significantly lower activity than before reaction (**Figure** **3**a). The deactivation process can help to understand the activity origin of OD‐Cu‐x. After the electrode was washed with ethanol and water and dried for further use, the current density remained poor, indicating the deactivation did not originate from the product or impurity covered on the surface of electrode. According to the SEM images, the OD‐Cu‐600 changed to a cube morphology with a reduced average size of 96.58 nm compared to the initial state, HRTEM images showed obvious lattice fringe corresponding to the Cu_2_O(111), while the Cu(111) was undetectable and the XRD further verified the formation of Cu_2_O (Figure S15, 16). The ECSA of OD‐Cu‐600 after reaction was about half of its original value (Figure S17), indicating a decrease in active sites. Further Cu LMM spectra of OD‐Cu‐600 after a one‐hour electrolysis test exhibited only the Cu^+^ peak, manifesting that the reaction process was accomplished with the oxidation of Cu^0^‐to‐Cu^+^ even though the anodic potential had not yet reached the onset potential for Cu^+^ formation (≈0.5V vs RHE) (Figure S18). When the OD‐Cu‐600 catalyst used in the single test was electroreduced under −0.4 V versus RHE, a large reduction current appeared, and the FFOR activity of OD‐Cu‐600 recovered. The above comparison indicated that the activity decay of OD‐Cu‐600 can be attributed to the dramatic oxidation of Cu^0^ sites on the OD‐Cu‐600 electrode, and the complete oxidation of Cu^0^ corresponded to the deactivation of FFOR. The XPS O 1s spectra of OD‐Cu‐600 after reaction under different potentials (0.2, 0.4, and 0.6 V vs RHE) were employed to explore the lattice oxygen transformation behavior. The proportion of lattice oxygen gradually increased with the positive shift of potential (11.7% under 0.2 V, 17.0% under 0.4 V, and 33.2% under 0.6 V vs RHE), and the transformation was accomplished with the increase and decline of FFOR activity, further confirming the lattice oxygen‐mediated FFOR activity.

The affinity and activation of substrate are usually considered important factors for the activity of heterogeneous catalysts.^[^ ^34^, ^35^ ^]^ Thus, the adsorption energy of FF molecule (E_ads_) on Cu(111) and Cu_2_O(111) was calculated. The adsorption energy of a single FF molecule on Cu(111) and Cu_2_O(111) was 0.64 eV and −0.53 eV, respectively, suggesting that the Cu^+^ sites were much better FF binding sites than Cu^0^, which was in contrast with the discussion above (Figure S20–21). Moreover, the open‐circuit potential drop tests exhibited that the OD‐Cu‐600, which represented the highest intrinsic activity among the series of OD‐Cu‐x catalysts, had the lowest adsorbed number of FF molecules in the outer Helmholtz layer (OHP) under the open‐circuit state. (Figure 2c and Figure S22).^[^ ^1^, ^36^ ^]^ These results suggested that the activity discrepancy of different OD‐Cu‐x catalysts did not arise from different Cu^0^/Cu^+^ ratios under open‐circuit conditions.

Intriguingly, the LSV curve of low‐potential FFOR on OD‐Cu‐x did not monotonically increase with the rise of potential as in conventional electrooxidation reactions.^[^ ^6^, ^34^, ^37^ ^]^ Instead, it was similar to an electrooxidation reaction catalyzed by metal that achieved the peaks at a certain potential, which were usually caused by the potential‐dependent surface species coverage.^[^ ^38^, ^39^ ^]^ As shown in Figure 3b, the three OD‐Cu catalysts all showed small oxidation peaks corresponding to the monolayer (OH)_ads_ formation under the potential of ≈0.37V vs RHE,^[^ ^40^, ^41^ ^]^ and the FFOR activity decreased sharply after this oxidation potential in Figure 2a. Based on the previous research and theoretical Pourbaix plot, the Cu^0^ was also covered by hydroxyl ((OH)_ads_) to form Cu(OH)_ads_ species before the oxidation peak of Cu^0^‐to‐Cu^+^ (Figure S23).^[^ ^22^, ^33^, ^42^, ^43^ ^]^ Thus, the surface species evolution under anodic potential can be hypothesized as the origin of FFOR activity, and the potential‐dependent (OH)_ads_ coverage can be a vital factor. The in‐situ Raman spectra under the oxidation potential showed a peak at $\approx 710\;{\text{cm}}^{-1}$, which confirmed the existence of Cu(OH)_ads_ species (Figure S24).^[^ ^42^, ^44^ ^]^ Further DFT calculations revealed that the Cu^+^ site near the residual lattice oxygen in OD‐Cu transformed to Cu(OH)_ads_ with a Δ*E* of −1.56 eV, suggesting a spontaneous transformation to form Cu(OH)_ads_, which was in agreement with previous literature.^[^ ^22^ ^]^ Instead, the Cu^0^‐to‐Cu(OH)_ads_ process on Cu(111) showed a Δ*E* of 0.80 eV, suggesting that the procedure should be driven under applied potential (Figure 3c, Figure S25). The operando electrochemical impedance spectra (EIS) of OD‐Cu‐600 under different potentials were also tested to verify the OH adsorption process (Figure S26).^[^ ^45^, ^46^ ^]^ The resistance of charge transfer (R_ct_) for Cu^0^ electrooxidation significantly decreased after the 0.4 V versus RHE. Based on the results, the Cu^0^ sites in OD‐Cu‐x catalysts will undergo an oxidation process accomplished with the OH covering and form the species of Cu(OH)_ads_.

Based on the comprehension of the real surface environment of OD‐Cu‐x catalysts during the electrooxidation process, the influence of varying (OH)_ads_ coverage on Cu(111) surface on the affinity of FF molecules was further explored. On the pristine Cu(111) surface (with zero (OH)_ads_), the E_ads_ was 0.64 eV. When the number of (OH)_ads_ increased to four, the most stable binding of FF with an E_ads_ of −0.64 eV appeared. With the increasing number of (OH)_ads_, the E_ads_ positively shifted and came to 1.24 eV on Cu(111) with eight (OH)_ads_, corresponding to a weaker interaction between FF molecule and Cu^0^ active site (Figure 3d, Figure S27–28). The projected density of states (pDOS) of Cu 3 d orbitals showed that the d‐band center (E_dbc_) positively shifted to the Fermi level, suggesting a gradually stronger binding of Cu(111) with FF (Figure S29). However, the increasing trend did not fully coincide with the volcano‐type relationship of E_ads_, suggesting that the change in E_ads_ was not solely controlled by the factor of electronic structure. Thus, the independent gradient model based on Hirshfeld partition (IGMH) was employed to investigate the weak interaction visually on the catalyst surface from the perspective of geometry structure and non‐covalent interaction. (Figure 3e)^[^ ^47^ ^]^ On the pristine Cu(111), the major interaction between the Cu(111) surface and FF molecule was the d–*π* overlap (the green region). When the (OH)_ads_ number came to four, the d‐*π* overlap still existed and an additional hydrogen bond between (OH)_ads_, the aldehyde group of FF appeared (the blue region), endowing the stronger binding intensity of FF. With the further increase of (OH)_ads_, the steric hindrance strengthened, and the d–*π* interaction was weakened; the main interaction was just the hydrogen bond, corresponding to the weak adsorption of FF. Based on the discussion above, the intrinsic activity difference of OD‐Cu‐x catalysts can also be understood. The OD‐Cu‐200 with abundant Cu^+^ sites endowed a high coverage of (OH)_ads_ under alkaline conditions, while the OD‐Cu‐1800 with insufficient Cu(OH)_ads_ sites weakened the adsorption and activation of FF molecules. Thus, the OD‐Cu‐600 with a moderate Cu^0^/Cu^+^ ratio balanced the coverage of (OH)_ads_ and furfural, realizing the optimal activity.

Despite the evolution of surface species and the adsorption of furfural molecules, the substrate activation behavior on Cu^0^ active sites can be another factor for FFOR activity. Thus, the interaction of furfural molecules and Cu^0^ sites at different applied potentials was examined with the grand canonical density functional theory (GC‐DFT).^[^ ^48^ ^]^ The planar‐average electron density represented that at the point of zero charge (PZC), the surface Cu(111) with a state of electron accumulation adsorbed the electron‐poor FF molecule, representing the electron transferred from FF to the surface of Cu(111) in the process of substrate adsorption. After the anodic potential was applied, the substrate activation behavior initially intensified but gradually became weaker as the potential shifted positively (Figure S30). The 2D contour plot of electron density suggested that the FF showed electron depletion property under the low potential window, which was beneficial for the nucleophilic attack by OH^−^ from electrolyte and subsequently promoted the kinetics of the overall reaction (Figure 3f). When applied at a more positive potential after 0.4 V versus RHE, the electrons accumulated on the FF, resulting in less reactivity and lower current density.

According to the previous analysis, for different OD‐Cu‐x catalysts, the valence state of Cu sites can modulate the coverage of surface hydroxyls under FFOR conditions and then endow the distinct intrinsic activity, the OD‐Cu‐600 with moderate ratio Cu^0^/Cu^+^ can be transformed to the surface with optimal coverage of (OH)_ads_, which bind FF molecules with d–*π* interaction and hydrogen bond simultaneously, promoting the mass transfer of substrate (Pathway I in Figure 3g). Moreover, the number of (OH)_ads_ for the same OD‐Cu‐x catalysts was also affected by applied potential; the (OH)_ads_ monolayer completely covered the Cu^0^ sites after ≈0.37V versus RHE, and the current density of low‐potential FFOR decreased sharply since the weak interaction between OD‐Cu‐x electrode and FF molecules. Thus, the selection of potential windows was also an effective method for obtaining adequate activation and rapid conversion of FF. (Pathway II in Figure 3g)

### 2.4 The Potential‐Dependent Anodic Hydrogen Evolution Pathway on OD‐Cu‐x Catalysts

**Figure 4:**
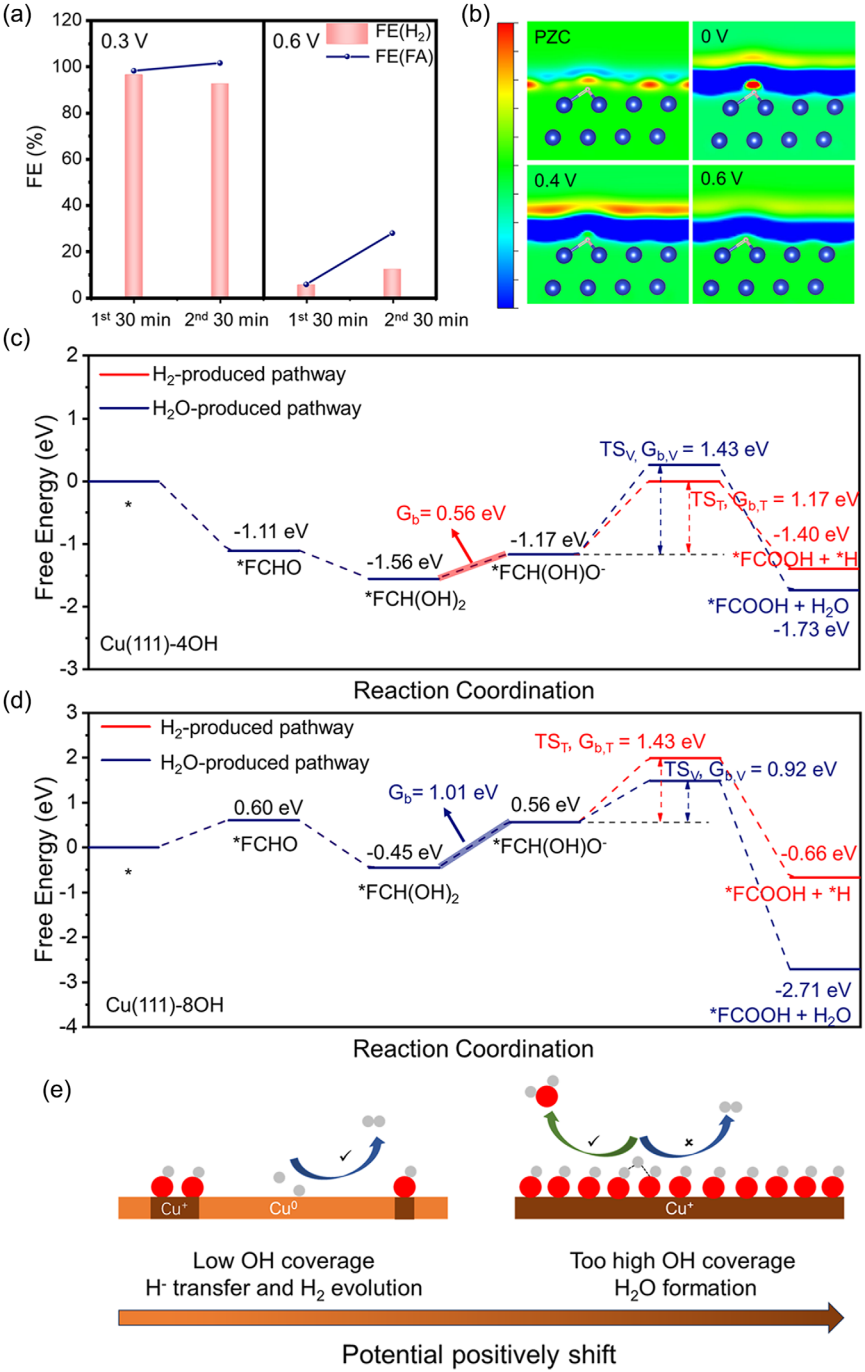
The potential‐dependent anodic H_2_ production pathway. a) The FE of FA and H_2_ in two separated 30‐minute electrolysis under 0.3 and 0.6 V versus RHE; b) the 2D contour plot of electron density for *H intermediate on Cu(111), the range of color bar is ±5 × 10^−3^ e Bohr^−3^; (c–d) the Gibbs free energy profiles of FFOR on Cu(111) with c) 4 OH and d) 8 OH; e) the illustration of distinct *H transfer processes on Cu catalyst with different OH coverages.

During the electrooxidation of FF, the H atom in the aldehyde group will detach and transfer to the surface of catalyst, then produce H_2_ via a Tafel pathway (Figure S31).^[^ ^13^, ^26^ ^]^ Thus, the Δ*G* of this HER process on Cu(111) and Cu_2_O(111) was calculated to investigate the site for HER. The Δ*G* on Cu(111) was −0.07 eV, a value of near zero, indicating its intrinsic property as the H_2_ evolution active site. By comparison, the Δ*G* of HER on Cu_2_O(111) was ‐0.45 eV, presenting a high barrier of H desorption (Figure S32). Thus, the series of OD‐Cu‐x catalysts with Cu^0^ sites all exhibited the ability to produce H_2_ on anode. However, the FE of H_2_ under the 0.3 V versus RHE on different OD‐Cu‐x catalysts exceeded 90% with hardly noteworthy difference, suggesting the hydrogen evolution process was not determined by the Cu^0^/Cu^+^ ratio. To further confirm the assertion, a one‐hour FFOR electrooxidation test was divided into two parts: the initial 30 min and the subsequent 30 min. The products of the two parts were quantified, and the FE was calculated separately (**Figure** **4**a, Figure S33). During the FFOR process, the Cu^0^ was gradually oxidized to Cu^+^. If the oxidation state of Cu played the major role in anodic H_2_ production, the FE would be significantly lower in the second half‐hour test. However, the FE of products both kept well in the different stages under the same potential, manifesting that the transfer of *H and HER process under the specific potential was not strongly associated with the oxidation state of Cu sites. According to the quantification of products in Figure 2d, the FE of anodically produced H_2_ kept near 100% in the low potential window, but dropped to <80% after 0.4 V versus RHE (related to monolayer (OH)_ads_ formation on the surface of OD‐Cu at the potential) and came to ≈0% after 0.5 V versus RHE (corresponding to the complete oxidation of Cu^0^). The phenomenon can be assigned to the reaction pathway switch from the Tafel pathway to a reverse Volmer pathway (*H + OH^−^ = H_2_O + e^−^),^[^ ^10^, ^27^ ^]^ and the switch was potential‐dependent.

Thus, the GC‐DFT was employed to explore the *H transfer and activation under the different potential windows (Figure 4b). At the PZC, the *H on Cu(111) exhibited a slight electron enrichment and the electron density further enlarged after a low anodic potential (<0.4 V vs RHE) was applied. Nevertheless, when the potential came to 0.4 V versus RHE, the electron density of *H lowered progressively and transformed to the H^+^ state, which tended to be attacked by OH^−^ from anolyte, resulting in the formation of H_2_O rather than H_2_ (Figure S34).

In addition to the intrinsic potential‐dependent *H activation property of Cu(111) surface, the effect of surface hydroxyl coverage was also taken into consideration. Based on the previously discussed difference in activity, the Cu(OH)_ads_ was the main form of Cu^+^ under the electrooxidation. Thus, the competition of the *H reaction pathway on different OH‐covered OD‐Cu was explored by calculating the Gibbs free energy barriers of Tafel step and reverse Volmer reaction (Figure S35–37). However, under the different coverage of OH, the reverse Volmer pathway was always the dominant *H transfer route, which was contrary to our experimental results under the low potential window. Therefore, we speculated that the coverage of *OH mainly affected the process of the H atom (which was identified as the origin of anode‐produced H_2_^[^ ^12^, ^33^, ^49^ ^]^ detachment from the aldehyde group of FF instead of the *H transfer, and the Gibbs free energy profile of overall reaction pathway was calculated (Figure 4e). The Cu(111) with 4 (OH)_ads_ showed a lower Δ*G* of −1.11 eV for the adsorption of FF than that of Cu(111) with 8 (OH)_ads_ (0.60 eV), indicating a strong affinity of FF molecules and consecutive spontaneous transfer, consistent with the adsorption behavior and kinetics analysis mentioned earlier. The Gibbs free energy barrier (G_b_) appeared in the step of gem diols intermediate (FCH(OH)_2_) to FCH(OH)O^−^, which can be identified as the potential‐dependent step (PDS). The G_b_ of Cu(111) with 4 (OH)_ads_ was 0.56 eV, while the value of Cu(111) with 8 (OH)_ads_ was 1.01 eV, suggesting that a lower limiting potential was needed on Cu(111) with 4 (OH)_ads_ for driving low‐potential FFOR.

The process of H detachment from intermediate *F‐CH(OH)O^−^ was the watershed to determine its evolution as H_2_ or its combination with OH to form H_2_O. On Cu(111) with 4 (OH)_ads_ and 8 (OH)_ads_, the processes of *H transfer to surface hydroxyl were both thermodynamically favored elementary steps. However, the *H can move to Cu^0^ sites with a lower activation energy barrier (G_b,T_ = 1.17 eV, T stood for the Tafel step) than that of binding with surface hydroxyl (G_b,V_ = 1.43 eV, V represented the reverse Volmer step) on Cu(111) with 4 (OH)_ads_, manifesting that the *H transferred to the Cu atom was kinetic‐preferred on the Cu(111) with low OH coverage. While on the surface of Cu(111) with 8 (OH)_ads_, the G_b, T_ was 1.43 eV and the G_b, V_ was 0.92 eV, suggesting that the *H easily captured by Cu(OH)_ads_ under the high (OH)_ads_ coverage, corresponding to a reverse Volmer step without anodic H_2_ production.

Through the comprehensive research mentioned earlier, the anodic H_2_ production was determined by potential‐dependent surface Cu(OH)_ads_ coverage and electron transfer behavior simultaneously. Under the low applied potential with the low OH convergence, the *H with electronegative property was able to transfer to the surface of Cu^0^ and form H_2_ via a Tafel pathway with a low energy barrier of 1.17 eV. With the gradual increase of OH convergence and electron depletion, the positively charged *H tended to be bound by surface OH and form H_2_O (Figure 4e). Thus, a potential window of 0–0.4 V versus RHE should be chosen to obtain value‐added product FA and H_2_ simultaneously with high utilization of the H atom in the aldehyde group, which agreed with experimental results (Figure 2d).

## 3 Conclusion

In summary, a series of OD‐Cu‐x catalysts with different Cu^0^/Cu^+^ ratios were successfully prepared, and detailed characterization of morphology and electronic structure confirmed the coexistence of Cu^0^ and Cu^+^ sites and the microenvironment with residual lattice oxygen in OD‐Cu‐x. When applied for the low‐potential FFOR, the as‐prepared OD‐Cu‐600 can achieve a low‐potential FF‐to‐FA electrooxidation process with anodic H_2_ production of only 0.081 V versus RHE at a current density of 10 mA cm^−2^, outperforming most state‐of‐art electrochemical FFOR catalysts. The FA and H_2_ can be produced on the anode with FE of 96.1% and 97.4%, respectively, in consecutive 10 cycles, exhibiting superior product selectivity and stability. Detailed in‐situ Raman spectra and DFT calculations confirmed that the Cu^+^ sites in OD‐Cu can be transformed to Cu(OH)_ads_ under FFOR conditions, and the coverage of Cu(OH)_ads_ was potential‐dependent. The theoretical simulation for reaction pathway of FFOR and electronic structure of OD‐Cu‐x catalysts indicated that the FF molecule adsorption behavior and the kinetics of *H transfer to Cu surface were both optimized by the Cu(OH)_ads_ coverage (25% based on DFT models), endowing the high FFOR activity with the H_2_ evolution from anode. The moderate Cu^0^/Cu^+^ ratio (83.3%) and suitable potential windows (0–0.4 V versus RHE) were both needed for highly active and atom‐economic co‐production of FA and H_2_ on the anode. This work offers a comprehensive insight into the potential‐dependent structure evolution of Cu‐based catalysts, as well as the related electrocatalytic kinetics and pathway of low‐potential FFOR.

## 4 Experimental Methods

4.1

4.1.1

##### Synthesis of Cu(OH)_*2*_

The Cu(OH)_2_ was prepared through an electrooxidation method. Commercial Cu foam (CF) was cut into pieces with a size of 1 × 3 cm and then washed with acetone, 1 M HCl, and deionized water using ultrasonics. A piece of dry CF was treated via the chronoamperometry (CP) method in a three‐electrode system. The CF, graphite rod, and Ag/AgCl (filling with 3 M KCl) electrode were used as the work electrode, counter electrode, and reference electrode, respectively. A 200‐s reduction process with a cathodic current density of 20 mA cm^−2^ was carried out to further guarantee the complete removal of the oxide layer on CF. Then, an anodic current density of 26 mA cm^−2^ was applied until the surface of CF entirely turned into deep blue. The as‐obtained Cu(OH)_2_/CF was washed with deionized water and dried in air for use.

##### Synthesis of Cu_*2*_ *O and OD‐Cu‐x*

The Cu_2_O was synthesized with a chemical reduction method. A piece of Cu(OH)_2_ was immersed in 1 M KOH with 1 M HCHO until: 1) the color turned from blue to brown and 2) the bubbles completely disappeared.

OD‐Cu‐x were obtained with in‐situ electroreduction of Cu_2_O. The structure of the three‐electrode system was the same as the electrooxidation process for preparing Cu(OH)_2_. The OD‐Cu‐x was prepared under −0.4 V versus RHE with × s (x = 100, 200, 400, 600, 800, 1800).

## Conflict of Interest

The authors declare no conflict of interest.

## Supporting information

Supplementary Material

## Data Availability

The data that support the findings of this study are available from the corresponding author upon reasonable request.
