# Supplementary material for: Potential‐Dependent Kinetics and Reaction Pathways of Low‐Potential Furfural Electrooxidation with Anodic H2 Production
Source: Small Sci. 2025 Jun 24;5(8):2500132. doi: 10.1002/smsc.202500132 (PMC12362779; doi:10.1002/smsc.202500132)
Supplement: Supplementary file 1 — Supplementary Material [file SMSC-5-2500132-s001.pdf]

Supplementary Information

Potential-dependent Kinetics and Pathway of Furfural Electrooxidation with Anodic H_2 Production

Zhaohui Wu,[a] Guihao Liu,[a] Ziheng Song,[a] Yihang Hu,[a] Tianqi Nie[a] and Yu-Fei Song*[a,b]

[a]State Key Laboratory of Chemical Resource Engineering, Beijing University of Chemical Technology, Beijing 100029 P. R. China. *E-mail: songyf@mail.buct.edu.cn

[b]Quzhou Institute for Innovation in Resource Chemical Engineering, Quzhou, Zhejiang Province, 324000 P. R. China.；

List of Contents:

Experimental Details

Results and Discussion

Figure S1. (1) The CP curve of electrooxidation process of Cu foam; (b) the CV curve of Cu foam in 1 M KOH.

Figure S2. The XRD patterns of Cu_2O, $Cu(OH)_2$, and Cu Foam with corresponding PDF.

Figure S3. The SEM image of Cu foam

Figure S4. The SEM images of (a) OD-Cu-200 and (b) OD-Cu-1800 and the HRTEM of (c) OD-Cu-200 and (d) OD-Cu-1800.

Figure S5. The XPS Cu 2p spectra of OD-Cu-x (x = 200, 600, 1800)

Figure S6. The XPS spectra of O 1s of (a) Cu_2O and (b) OD-Cu-x (x=200, 600, 1800).

Figure S7. (a) The LSV curves and (b) ECSA normalized LSV curves of OD-Cu-x (x = 200, 600, 1800) and Cu foam.

Figure S8. The ECSA and C_{dl} measurement of OD-Cu-x catalysts.

Figure S9. (a) The LSV of OD-Cu-x catalysts and (b) the LSV normalized with ECSAs.

Figure S10. The HPLC plots of mix sample with different concentrations of FF, FA, and FL; (b-d) calibration curve of (b) FF, (c) FA, and (d) FL.

Figure S11. (a) The yield rate of FA and FL and (b) the yield rate of FA and H_2 on OD-Cu-600 under different potentials

Figure S12. The GC spectrum of the gas product under 0.5 V vs. RHE

Figure S13. The ^{1}H-NMR spectrum of the electrolyte under 0.5 V vs. RHE

Figure S14. The SCN^- poison experiments of OD-Cu-600.

Figure S15. (a-b) The SEM of OD-Cu-600 after reaction, the inset was the size distribution of OD-Cu-600 after reaction; (c) the HRTEM and (d) lattice fringe of OD-Cu-600 after reaction.

Figure S16. The XRD of OC-Cu-600 after reaction under 0.3 V *vs.* RHE

Figure S17. The C_{dl} of OD-Cu-600 catalysts before and after reaction.

Figure S18. (a) The Cu LMM spectra of OD-Cu-600 before and after reaction under 0.3 V *vs.* RHE; (b) the Cu 2p spectrum after reaction

Figure S19. The XPS O 1s after reaction under different potentials.

Figure S20. The adsorption energy of FF on Cu(111) and Cu_2O(111).

Figure S21. The optimized furfural adsorption configurations on (a) Cu(111) and (b) Cu_2O(111).

Figure S22. The OCP drop experiments of OD-Cu-x catalysts (x = 200, 600, 1800).

Figure S23. The calculated equilibrium Pourbaix plot of Cu.[1]

Figure S24. The *in-situ* Raman spectra of OD-Cu-600 during low-potential FFOR.

Figure S25. The optimized configuration of (a) before and (b) after OH adsorption on Cu(111) and on (c-d) OD-Cu(111).

Figure S26. (a) The operando EIS of OD-Cu-600 and (b) the R_{ct} fitting results.

Figure S27. The optimized models of Cu(111) with different OH coverages.

Figure S28. The optimized FF adsorption configurations of Cu(111) with different OH coverages.

Figure S29. The pDOS of Cu 3d orbitals in Cu(111) with different OH coverages.

Figure S30. The plane-average electron density of FF-adsorbed Cu(111).

Figure S31. The reaction pathway of FF low-potential oxidation on Cu catalysts.

Figure S32. The free energy profile of HER on Cu(111) and Cu_2O(111).

Figure S33. The i-t curves of 30-min electrolysis under 0.3 V and 0.6 V *vs.* RHE, respectively.

Figure S34. The plane-average electron density of Cu(111).

Figure S35. The optimized H adsorption configurations on Cu(111) with different OH coverages.

Figure S36. The optimized H_2O adsorption configurations on Cu(111) with different OH coverages.

Figure S37. The energy barrier comparison of Tafel step and reverse Volmer step on Cu(111) with different OH coverages.

Table S1. The fitting results for Cu LMM spectra of OD-Cu-x (x=200, 600, 1800)

Table S2. The fitting results for XPS O 1s spectra of OD-Cu-x (x=200, 600, 1800)

Table S3. Comparison of the electrocatalytic activity of OD-Cu-600 with recently reported transition metal-based MOR electrocatalysts in alkaline media.

Table S4. The fitting results for XPS O 1s spectra of OD-Cu-600 after different potentials

Table S5. The fitting results of operando EIS of OD-Cu-600 in 1 M KOH under different potentials

Experimental Details

Materials

KOH (> 95%, Macklin), formaldehyde aqueous solution (37~40 wt% HCHO, Xilong), furfural (99%, Energy Chemicals). All chemicals were used directly without further purification. Deionized water was used in all the experimental processes. Nafion 117 membrane was purchased from Dupont.

Characterization

X-ray diffraction (XRD) patterns were tested on a Bruker D8 diffractometer with Cu-Kα radiation. Scanning electron microscopy (SEM) images were recorded on the Zeiss Supra 55 SEM. High-resolution transmission electron microscopy (HRTEM) images were collected on a JEOL JEM-2010 electron microscope operating at 200 kV. X-ray photoelectronic spectroscopy was measured with. In-situ Raman spectra were characterized by the LabRAM Aramis (HORIBA Jobin Yvon S.A.S, France), the laser was 532 nm.

Electrochemical measurements

The electrochemical performances were tested in a three-electrode H-type cell by an electrochemical workstation (CHI 660e, CH, Shanghai). Graphite rod and Ag/AgCl electrode were used as counter and reference electrode, respectively.

The linear sweep voltammetry (LSV) curves were collected at a scan rate of 5 mV s^{-1}. AC impedance measurements were carried out in the same configuration from 0.01 Hz to 100 kHz with an AC voltage of 5 mV. The current density differences (Δj = ja-jc) were plotted against scan rates, and the linear slope is twice the double-layer capacitance (C_{dl}), and the ECSA can be calculated using the following equation: ECSA $=S_{geo}*C_{dl}/C_s$, where S_{geo} represents the geometric surface area of the working electrode (1 cm^2) and C_s is specific electrochemical double-layer capacitance and the value in alkaline media is 0.040 mF cm^{-2}.

Product Detection and Quantification

The gas products were quantified by Shimadzu GC-2014 gas chromatograph (equipped with FID and TCD detectors). The liquid product content of the anodic electrolytes was determined

with high-performance liquid chromatography (HPLC, Agilent 1260), which was equipped with Agilent 5HC-C18 column and variable wavelength detector (VWD). The mobile phase was methanol and 5 mM ammonium formate aqueous solution (v/v = 3:7), the flow rate was 0.6 mL min^{-1}

The selectivity and FE of products was calculated as follows:

$$Selectivity\ (FA) = \frac{n(FA)}{n(All\ product\ of\ FFOR)}$$

$$FE\ (FA) = \frac{1n(FA)}{n(All\ product\ of\ FFOR)}$$

$$FE\ (H_2) = \frac{n(H_2) * F * 2e^-}{Q_{total}}$$

The n(FA) was corrected since the inevitable Cannizzaro reaction

Computational Details

All spin-polarized DFT calculations were performed using the QUICKSTEP module in the CP2K package (version 2023.1)[2]. The exchange-correlation potential in the single-electron Kohn-Sham equation was described by generalized gradient approximation (GGA) with the Perdew-Burke-Ernzerhof (PBE) functional[3]. Wavefunctions were expanded in molecularly optimized (MOLOPT) double ζ-valence polarized basis set[4] with an auxiliary plane-wave basis set with a cutoff energy of 400 Ry. Core electrons were modeled by scalar relativistic norm-conserving Goedecker-Teter-Hutter (GTH) pseudopotentials[5] with 14, 4, 6, and 1 valence electrons for Cu, C, O, and H, respectively. Grimme's D3 method with Becke-Johnson (B-J) damping [6, 7] was added to study the impact of van der Waals interaction. The Monkhorst-Pack gamma centered 3×3×1 k point mesh was used for Brillouin zone sampling during geometry optimization process and self-consistent field (SCF) interaction was carried out with a conventional diagonalized method. When calculate the adsorption energy of small molecules, the exchange-correlation functional was changed to BEEF-vdW[8], which has been proven as a state-of-art functional to exactly calculate adsorption energy on the transition metal surfaces[9]. The transition state was searched with dimer method.[10]

The density of states (DOS) was analyzed with Multiwfn package[11] via the formed molden file in single energy point calculations. 60 empty orbitals were added to calculated the unoccupied

states. The projected DOS (pDOS) was calculated with the C-Squared Population Analysis (SCPA) method[12] and the Gaussian function was selected to be the broadening function.

The visual weak interaction analysis was carried out with Multiwfn package. The two fragments in Independent gradient model based on Hirshfeld partition (IGMH) analysis [13] was defined as hydrogenate Cu slab and furfural molecule, respectively.

The JDFTx software[14] was employed to examine the electron transfer behavior under different potentials based on the optimized structure by CP2K. The charge-asymmetric nonlocally determined local-electric (CANDLE) model proposed by Goddard et al[15] was used to take solvent environment into consideration. The cation and anion were selected as 1 M K^+ and 1 M F^-, respectively.

Results and Discussion

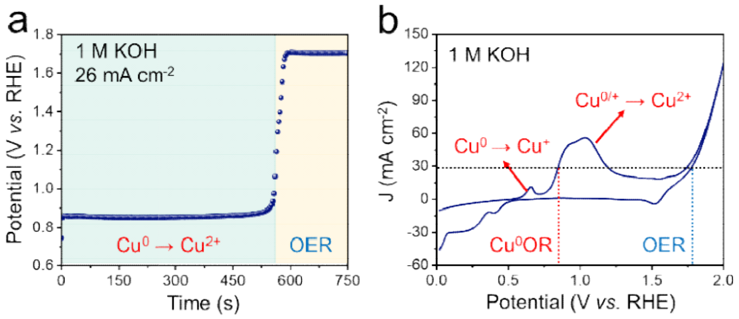

Figure S1. (1) The CP curve of electrooxidation process of Cu foam; (b) the CV curve of Cu foam in 1 M KOH.

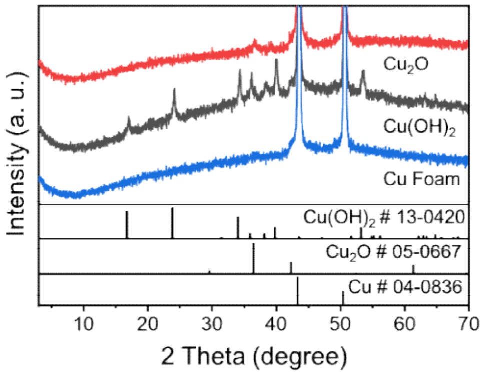

Figure S2. The XRD patterns of Cu_2O, $Cu(OH)_2$, and Cu Foam with corresponding PDF.

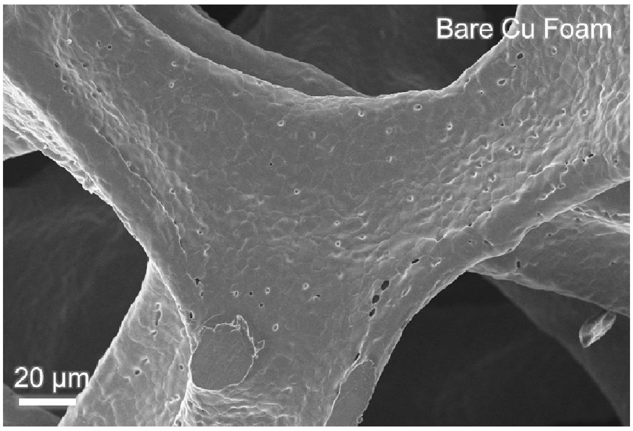

Figure S3. The SEM image of Cu foam

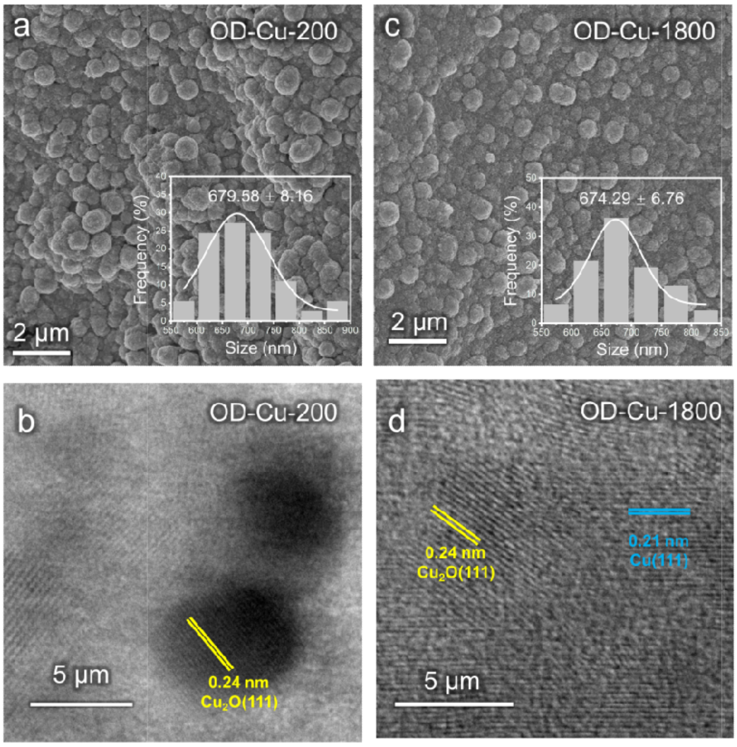

Figure S4. The SEM images of (a) OD-Cu-200 and (b) OD-Cu-1800 and the HRTEM of (c) OD-Cu-200 and (d) OD-Cu-1800.

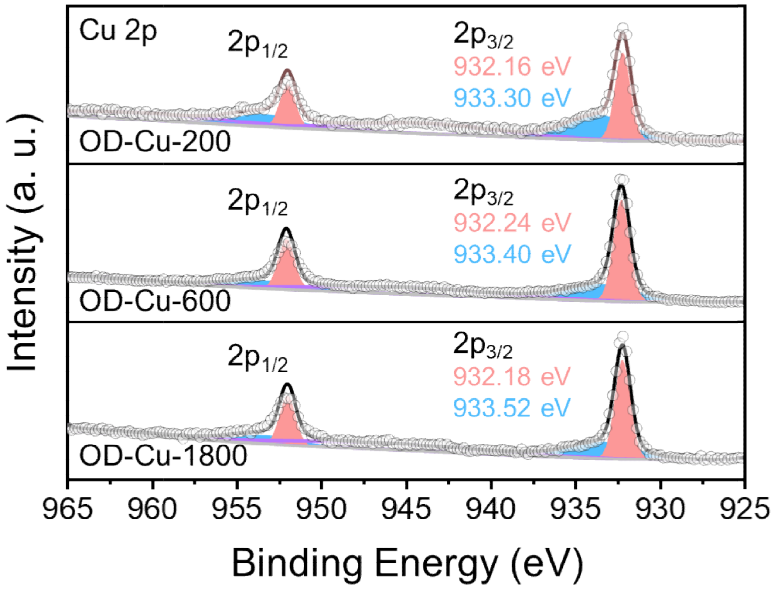

Figure S5. The XPS Cu 2p spectra of OD-Cu-x (x = 200, 600, 1800)

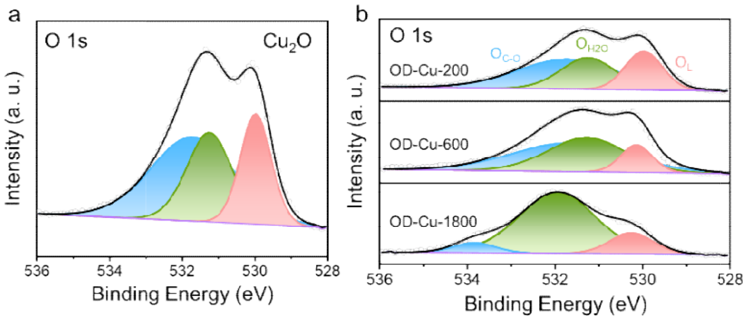

Figure S6. The XPS spectra of O 1s of (a) Cu_2O and (b) OD-Cu-x (x=200, 600, 1800).

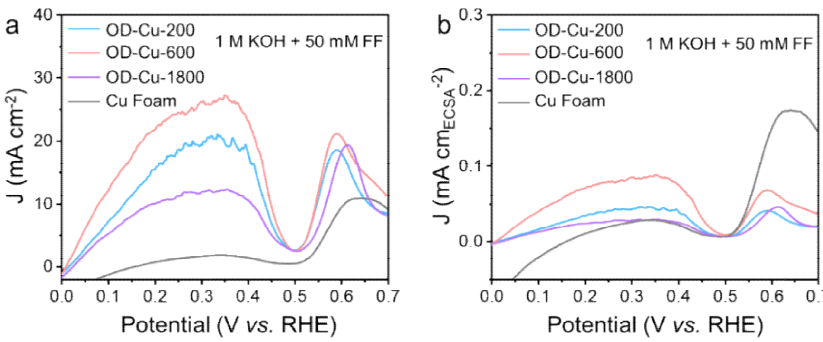

Figure S7. (a) The LSV curves and (b) ECSA normalized LSV curves of OD-Cu-x (x = 200, 600, 1800) and Cu foam.

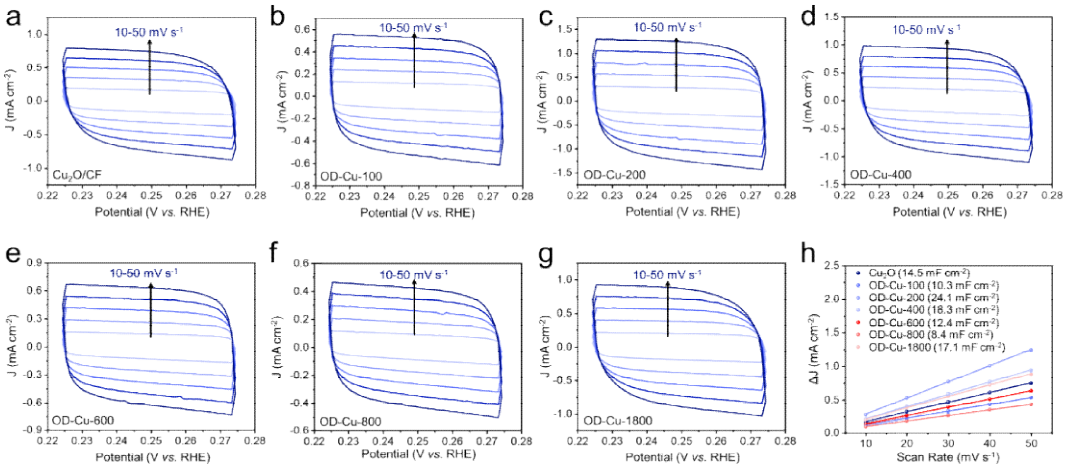

Figure S8. The ECSA and C_{dl} measurement of OD-Cu-x catalysts.

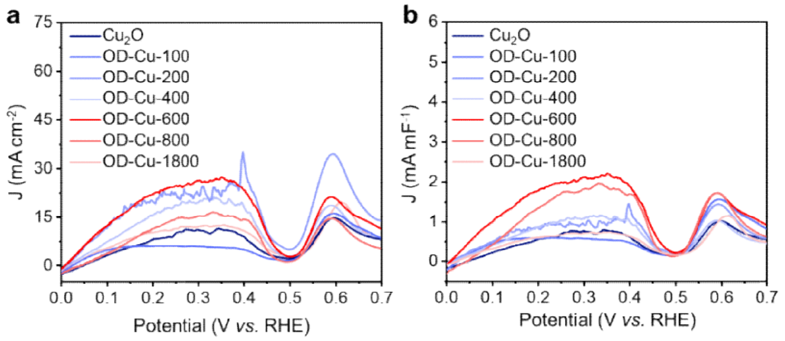

Figure S9. (a) The LSV of OD-Cu-x catalysts and (b) the LSV normalized with ECSAs.

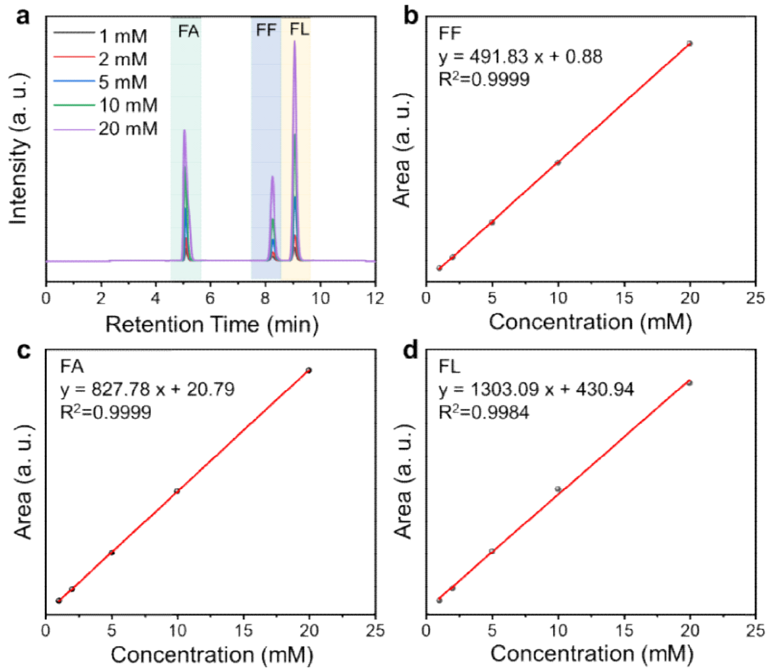

Figure S10. The HPLC plots of mix sample with different concentrations of FF, FA, and FL; (b-d) calibration curve of (b) FF, (c) FA, and (d) FL.

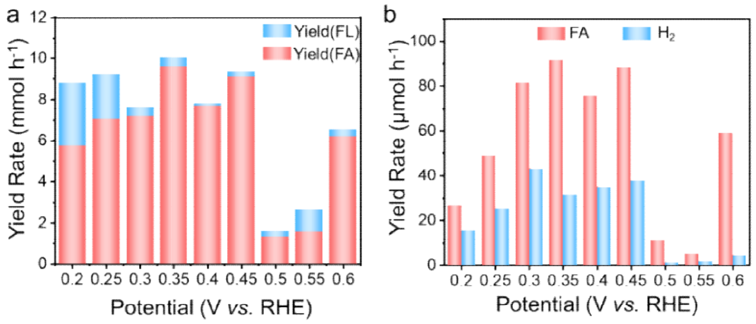

Figure S11. (a) The yield rate of FA and FL and (b) the yield rate of FA and H_2 on OD-Cu-600 under different potentials

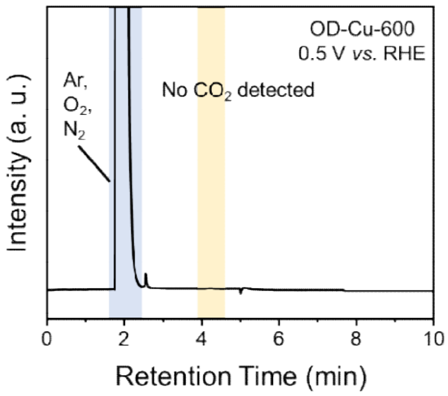

Figure S12. The GC spectrum of the gas product under 0.5 V *vs.* RHE

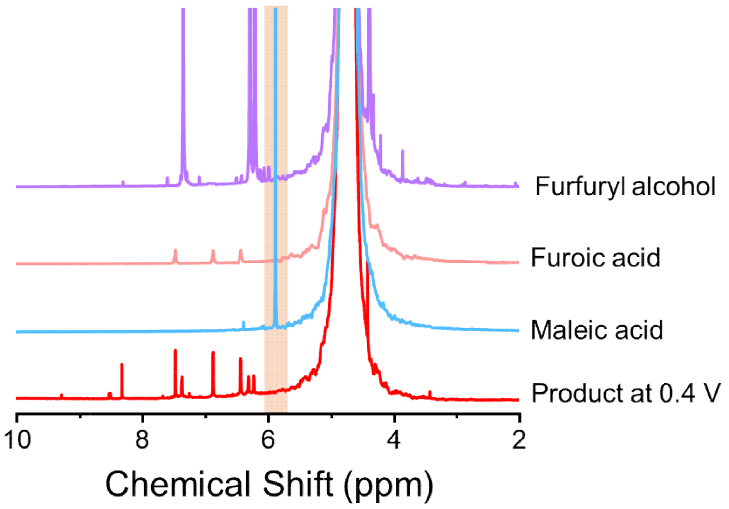

Figure S13. The ^{1}H-NMR spectrum of the electrolyte under 0.5 V vs. RHE

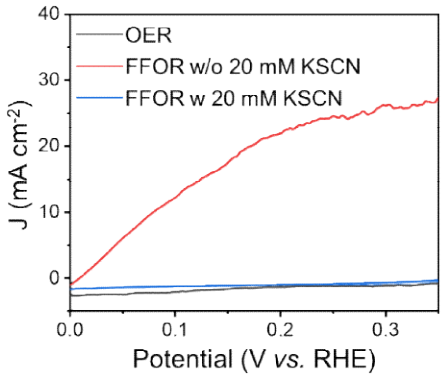

Figure S14. The SCN^- poison experiments of OD-Cu-600.

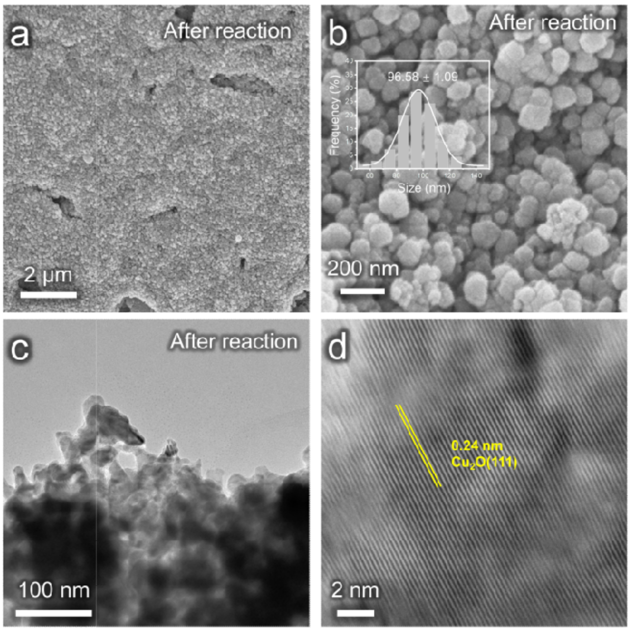

Figure S15. (a-b) The SEM of OD-Cu-600 after reaction, the inset was the size distribution of OD-Cu-600 after reaction; (c) the HRTEM and (d) lattice fringe of OD-Cu-600 after reaction.

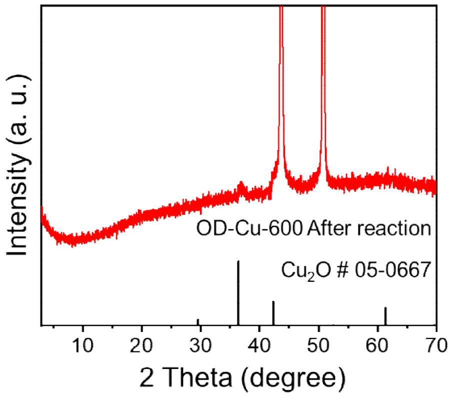

Figure S16. The XRD of OC-Cu-600 after reaction under 0.3 V *vs.* RHE

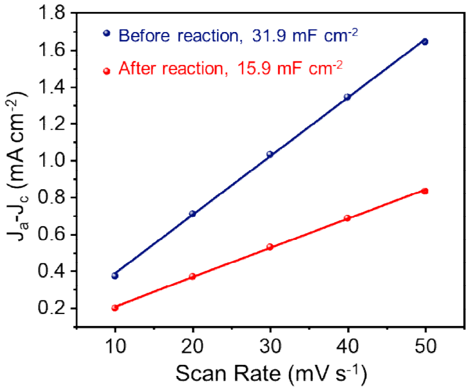

Figure S17. The C_{dl} of OD-Cu-600 catalysts before and after reaction.

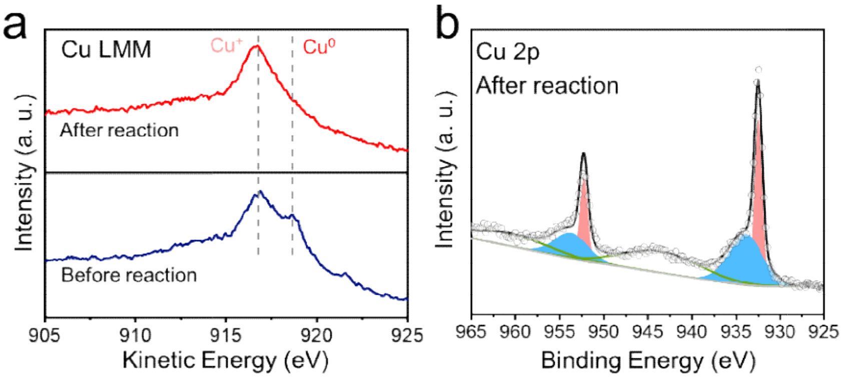

Figure S18. (a) The Cu LMM spectra of OD-Cu-600 before and after reaction under 0.3 V *vs.* RHE; (b) the Cu 2p spectrum after reaction

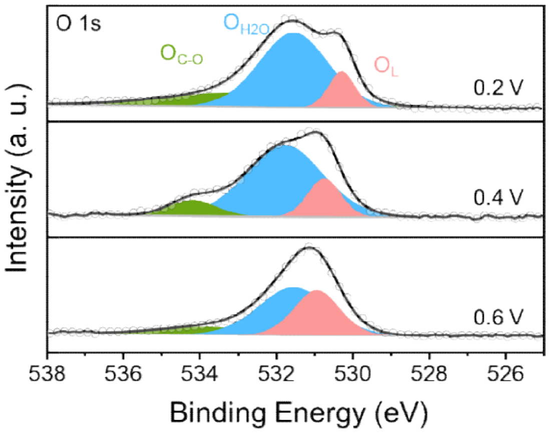

Figure S19. The XPS O 1s after reaction under different potentials.

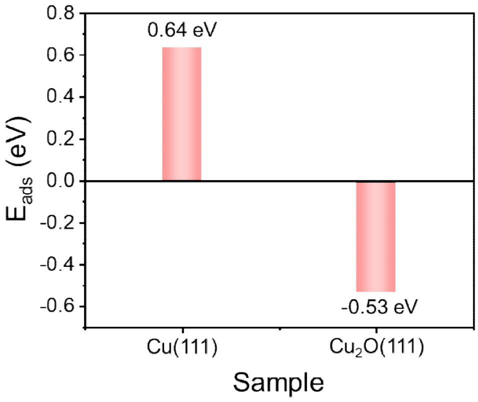

Figure S20. The adsorption energy of FF on Cu(111) and $Cu_2O(111)$.

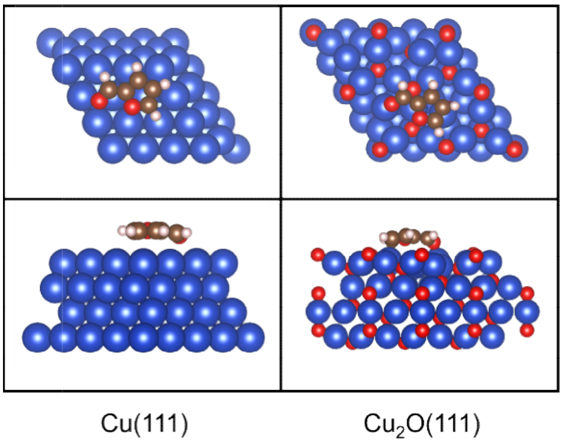

Figure S21. The optimized furfural adsorption configurations on (a) Cu(111) and (b) $Cu_2O(111)$.

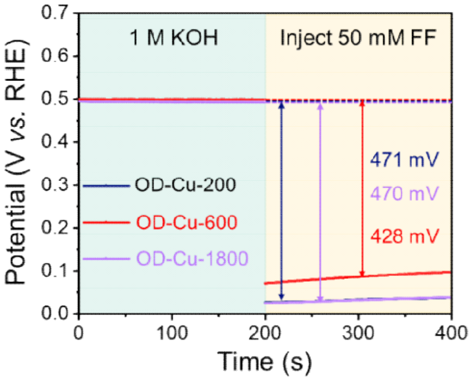

Figure S22. The OCP drop experiments of OD-Cu-x catalysts (x = 200, 600, 1800).

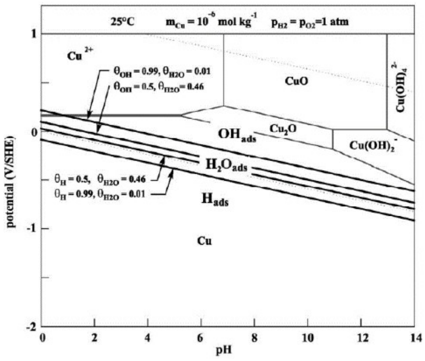

Figure S23. The calculated equilibrium Pourbaix plot of Cu.[1]

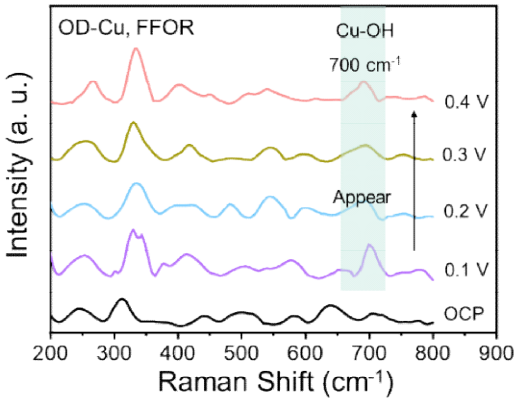

Figure S24. The *in-situ* Raman spectra of OD-Cu-600 during low-potential FFOR.

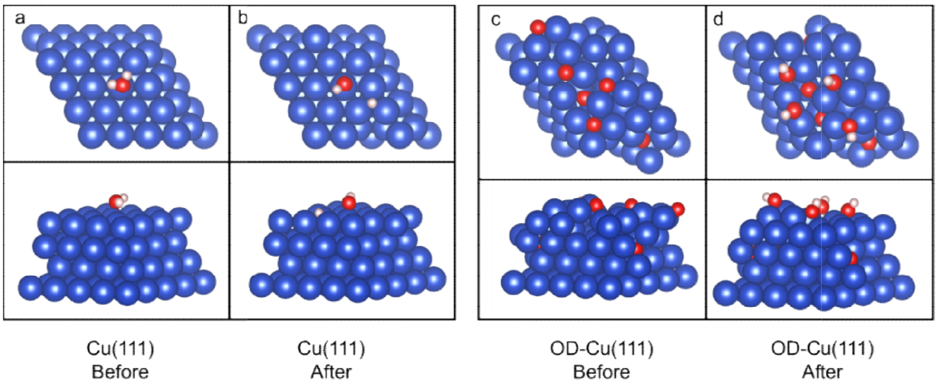

Figure S25. The optimized configuration of (a) before and (b) after OH adsorption on Cu(111) and on (c-d) OD-Cu(111).

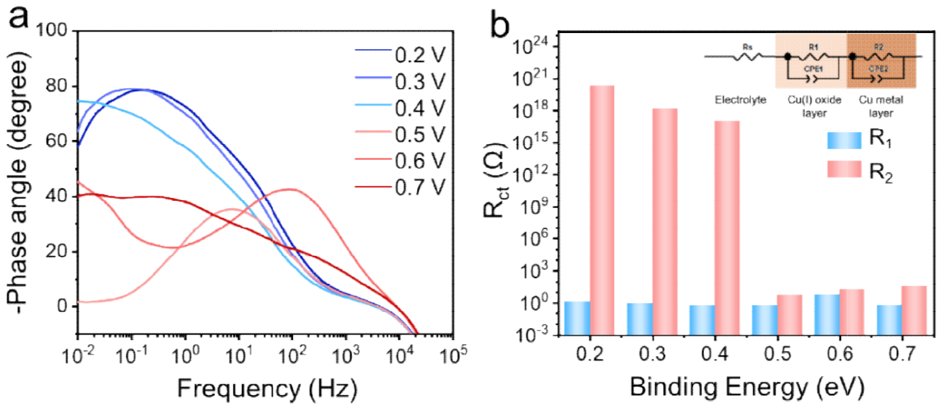

Figure S26. (a) The operando EIS of OD-Cu-600 and (b) the R_{ct} fitting results.

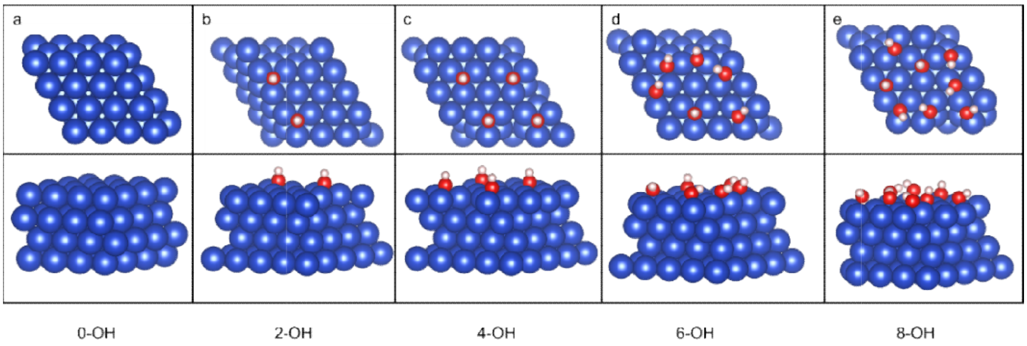

Figure S27. The optimized models of Cu(111) with different OH coverages.

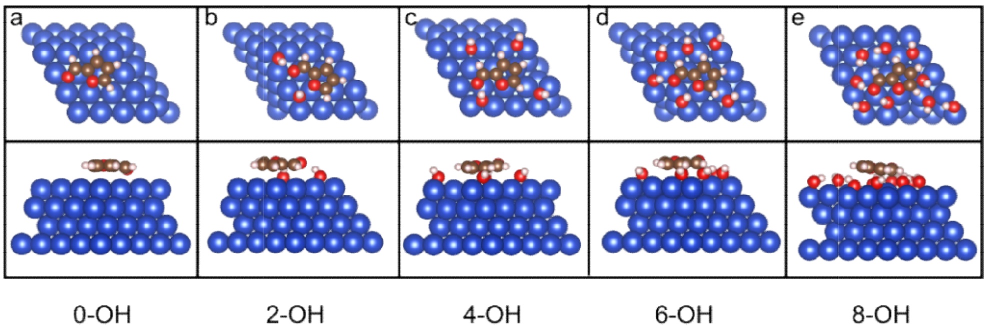

Figure S28. The optimized FF adsorption configurations of Cu(111) with different OH coverages.

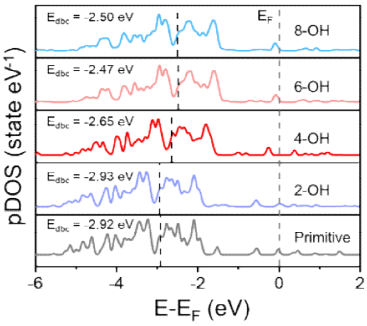

Figure S29. The pDOS of Cu 3d orbitals in Cu(111) with different OH coverages.

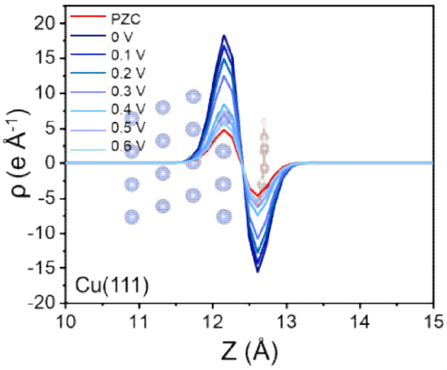

Figure S30. The plane-average electron density of FF-adsorbed Cu(111).

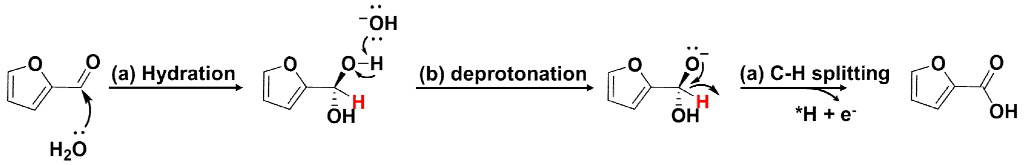

Figure S31. The reaction pathway of FF low-potential oxidation on Cu catalysts.

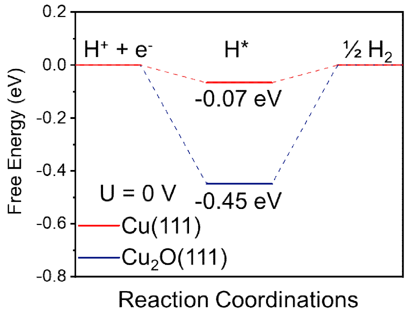

Figure S32. The free energy profile of HER on Cu(111) and $Cu_2O(111)$.

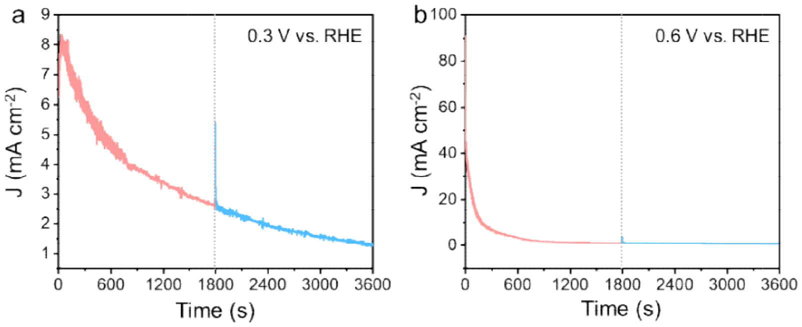

Figure S33. The i-t curves of 30-min electrolysis under 0.3 V and 0.6 V *vs.* RHE, respectively.

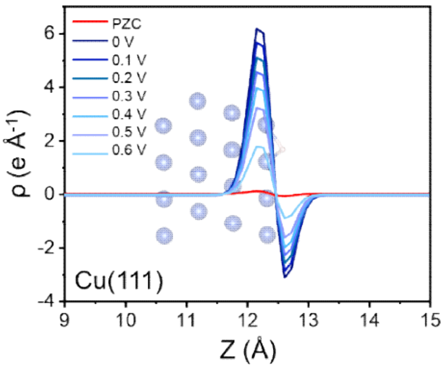

Figure S34. The plane-average electron density of Cu(111).

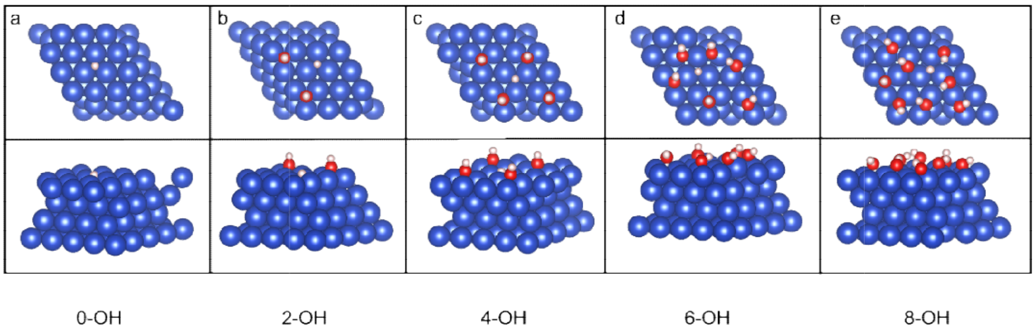

Figure S35. The optimized H adsorption configurations on Cu(111) with different OH coverages.

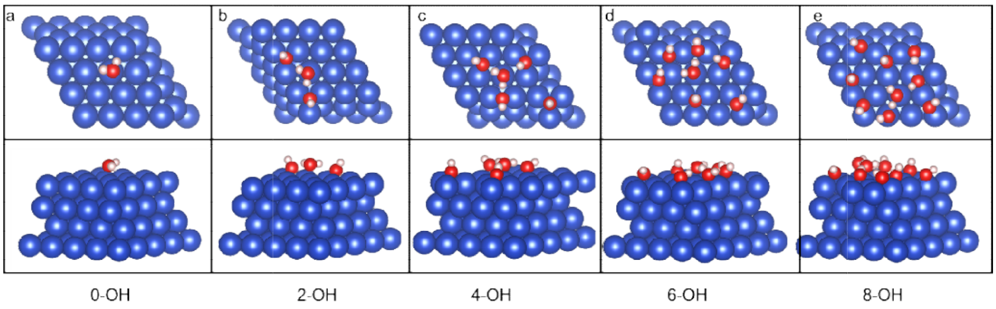

Figure S36. The optimized H_2O adsorption configurations on Cu(111) with different OH coverages.

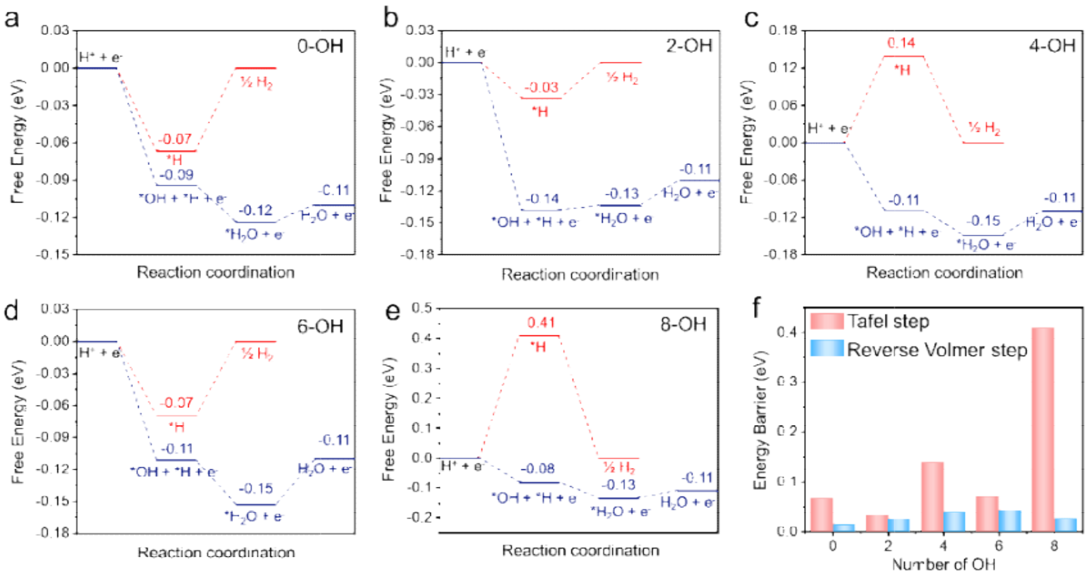

Figure S37. The energy barrier comparison of Tafel step and reverse Volmer step on Cu(111) with different OH coverages.

Table S1. The fitting results for Cu LMM spectra of OD-Cu-x (x=200, 600, 1800)

Sample	Area (Cu^0)	Area (Cu^+)	Ratio of Cu^0/Cu^+
**OD-Cu-200**	2080.8	8659.1	0.240
**OD-Cu-600**	7924.5	9515.1	0.833
**OD-Cu-1800**	28233.6	29980.2	0.942

Table S2. The fitting results for XPS O 1s spectra of OD-Cu-x (x=200, 600, 1800)

Sample	Area (O_{C-O})	Area (O_{H2O})	Area (O_L)	Ratio of O_L
**Cu_2O**	22413.8	20907.5	15954.4	0.269
**OD-Cu-200**	10853.6	5723.8	3953.5	0.192
**OD-Cu-600**	22697.9	15883.3	6999.4	0.154
**OD-Cu-1800**	2688.1	30120.1	5829.4	0.151

Table S3. Comparison of the electrocatalytic activity of OD-Cu-600 with recently reported transition metal-based MOR electrocatalysts in alkaline media.

Catalyst	Substrate (Electrolyte)	Anodic Product	J (mA cm^{-2}) @ E(V *vs.* RHE)	Selectivity / FE	Reference
OD-Cu-600	50 mM FF (1 M KOH)	FA, H_2	10@0.081	97%	This work
Rh_1Cu	30 mM FF (1 M KOH)	FA, H_2	10@0.031	~77%	Angew. Chem. Int. Ed. 2023, e202304852
MV-Cu	50 mM FF (1 M KOH)	FA, H_2	10@0.035	99%	Adv. Mater., 2023, 2304203
Cu/Cu Foam	50 mM FF (1 M KOH)	FA, H_2	10@0.076	~100 %	Angew. Chem. Int. Ed. 2022, e202115636
$CuAg_{glv}/Cu$	200 mM FF (1 M KOH)	FA, H_2	10@0.19	~94%	Energy Environ. Sci., 2022, 15, 4175
H-PdCu Ans	100 mM FF (0.1 M KOH)	FA, H_2	10@~0.32	N/A[a]	Appl Catal B-Environ, 2023, 328, 122530
Ag_2O/Ni Foam	50 mM FF (1 M KOH)	FA, H_2	100@1.32	~45%	J. Energy Chem.,2023, 79, 135

a: no mentioned in the literature

Table S4. The fitting results for XPS O 1s spectra of OD-Cu-600 after different potentials

Potential	Area (O_{C-O})	Area (O_{H2O})	Area (O_L)	Ratio of O_L
**0.2 V**	5983.9	14287.4	2683.0	0.117
**0.4 V**	2174.0	15857.1	3682.9	0.170
**0.6 V**	3661.9	14782.2	9180.4	0.332

Table S5. The fitting results of operando EIS of OD-Cu-600 in 1 M KOH under different potentials

Potential (V *vs.* RHE)	Rs (Ω)	R_1 (Ω)	CPE_1-T (S s^n cm^{-2})	CPE_1-P	R_2 (Ω)	CPE_2-T(S s^n cm^{-2})	CPE_2-P
**0.2**	0.66639	1.065	0.044548	0.7728	2.237E20	0.016225	0.87889
**0.3**	0.66191	0.8183	0.060026	0.76474	1.608E18	0.021965	0.85899
**0.4**	0.63517	0.43466	0.11814	0.80598	9.824E16	0.04814	0.73936
**0.5**	0.63237	0.50895	0.047272	0.79798	5.077	0.034361	0.79189
**0.6**	0.6685	5.706	0.0034645	0.79698	15.71	0.014205	0.759

**0.7**	0.68909	0.53325	0.011214	0.77122	31.42	0.055369	0.58943

References

1. E. Protopopoff; P. Marcus, *Electrochim. Acta* **2005,** *51*, 408.
2. T. D. Kühne; M. Iannuzzi; M. D. Ben; V. V. Rybkin; P. Seewald; F. Stein; T. Laino; R. Z. Khaliullin; O. Schütt; F. Schiffmann; D. Golze; J. Wilhelm; S. Chulkov; M. H. Bani-Hashemian; V. Weber; U. Borštnik; M. Taillefumier; A. S. Jakobovits; A. Lazzaro; H. Pabst; T. Müller; R. Schade; M. Guidon; S. Andermatt; N. Holmberg; G. K. Schenter; A. Hehn; A. Bussy; F. Belleflamme; G. Tabacchi; A. Glöß; M. Lass; I. Bethune; C. J. Mundy; C. Plessl; M. Watkins; J. VandeVondele; M. Krack; J. Hutter, *J. Chem. Phys.* **2020,** *152*, 194103.
3. J. P. Perdew; K. Burke; M. Ernzerhof, *Phys. Rev. Lett.* **1996,** *77*, 3865.
4. J. VandeVondele; J. Hutter, *J. Chem. Phys.* **2007,** *127*, 114105.
5. C. Hartwigsen; S. Goedecker; J. Hutter, *Phys. Rev. B* **1998,** *56*, 3641.
6. S. Grimme; J. Antony; S. Ehrlich; H. Krieg, *J. Chem. Phys.* **2010,** *132*, 154104.
7. S. Grimme; S. Ehrlich; L. Goerigk, *J. Comput. Chem.* **2011,** *32*, 1456.
8. J. Wellendorff; K. T. Lundgaard; A. Møgelhøj; V. Petzold; D. D. Landis; Jens K. Nørskov; T. Bligaard; K. W. Jacobsen, *Phys. Rev. B* **2012,** *85*, 235149.
9. J. Wellendorff; T. L. Silbaugh; D. Garcia-Pintos; J. K. Nørskov; T. Bligaard; F. Studt; C. T. Campbell, *Surf. Sci.* **2015,** *640*, 36.
10. G. Henkelman; H. Jónsson, *J. Chem. Phys.* **1999,** *111*, 7010.
11. T. Lu; F. Chen, *J. Comput. Chem.* **2012,** *33*, 580.
12. P. Ros; A. V. D. Avoird; G. C. A. Schuit, *Coord. Chem. Rev.* **1967,** *2*, 77.
13. T. Lu; Q. Chen, *J. Comput. Chem.* **2022,** *43*, 539.
14. R. Sundararaman; K. Letchworth-Weaver; K. A. Schwarz; D. Gunceler; Y. Ozhabes; T. A. Arias, *SoftwareX* **2017,** *6*, 278.
15. R. Sundararaman; W. A. G. III, *J. Chem. Phys.* **2015,** *142*, 064107.
